# A Drug Repurposing Approach for Antimalarials Interfering with SARS-CoV-2 Spike Protein Receptor Binding Domain (RBD) and Human Angiotensin-Converting Enzyme 2 (ACE2)

**DOI:** 10.3390/ph14100954

**Published:** 2021-09-23

**Authors:** Paolo Coghi, Li Jun Yang, Jerome P. L. Ng, Richard K. Haynes, Maurizio Memo, Alessandra Gianoncelli, Vincent Kam Wai Wong, Giovanni Ribaudo

**Affiliations:** 1 School of Pharmacy, Macau University of Science and Technology, Taipa 999078, China; coghips@must.edu.mo; 2 Neher’s Biophysics Laboratory for Innovative Drug Discovery, State Key Laboratory of Quality Research in Chinese Medicine, Macau University of Science and Technology, Taipa 999078, China; lijunyang224@163.com (L.J.Y.); jeromeplng@outlook.com (J.P.L.N.); 3 Center of Excellence for Pharmaceutical Sciences, Faculty of Health Sciences, North-West University Potchefstroom, Potchefstroom 2531, South Africa; haynes@ust.hk; 4 Department of Molecular and Translational Medicine, University of Brescia, 25121 Brescia, Italy; maurizio.memo@unibs.it (M.M.); alessandra.gianoncelli@unibs.it (A.G.)

**Keywords:** SARS-CoV-2, spike protein, RBD, molecular dynamics, bio-layer interferometry, antimalarial drugs, artemisone, pyronaridine, drug repurposing

## Abstract

Host cell invasion by severe acute respiratory syndrome coronavirus 2 (SARS-CoV-2) is mediated by the interaction of the viral spike protein (S) with human angiotensin-converting enzyme 2 (ACE2) through the receptor-binding domain (RBD). In this work, computational and experimental techniques were combined to screen antimalarial compounds from different chemical classes, with the aim of identifying small molecules interfering with the RBD-ACE2 interaction and, consequently, with cell invasion. Docking studies showed that the compounds interfere with the same region of the RBD, but different interaction patterns were noted for ACE2. Virtual screening indicated pyronaridine as the most promising RBD and ACE2 ligand, and molecular dynamics simulations confirmed the stability of the predicted complex with the RBD. Bio-layer interferometry showed that artemisone and methylene blue have a strong binding affinity for RBD (K_D_ = 0.363 and 0.226 μM). Pyronaridine also binds RBD and ACE2 in vitro (K_D_ = 56.8 and 51.3 μM). Overall, these three compounds inhibit the binding of RBD to ACE2 in the μM range, supporting the in silico data.

## 1. Introduction

Severe acute respiratory syndrome coronavirus 2 (SARS-CoV-2) is the cause of the ongoing coronavirus disease 2019 (COVID-19) pandemic. Despite containment measures, infections by SARS-CoV-2 present a formidable challenge for healthcare systems worldwide. The infection results in respiratory symptoms such as a cough and shortness of breath, and may develop into severe pneumonia with associated acute respiratory distress syndrome (ARDS) [1]. Although SARS-CoV-2 vaccines are available, the quest for small molecules that may effectively be used to treat the viral infection is an urgent and ongoing task [2]. In this context, drug repurposing represents an attractive strategy. The caveat though is that such drugs must definitively act on the virus, and not as agents that induce phospholipidosis or other off-target effects [3].

During its replication, the viral genome is transcribed into structural proteins of the envelope (E), membrane (M), nucleocapsid (N) and spike (S), which is composed of domains S1 and S2, as well as of non-structural and accessory proteins (ORFs) [4,5,6]. The angiotensin-converting enzyme 2 (ACE2) is the main human receptor of SARS-CoV, which interacts with the host cell through the S1 domain [7]. The S1 subunit contains a receptor-binding domain (RBD), which directly mediates this binding, and an N-terminal domain, the same motif that is also present for SARS-CoV-2 [8]. The identification of small molecule inhibitors targeting these viral proteins, host proteins or RNA assemblies unique to SARS-CoV-2 are thus required [9,10]. In this connection, several of these targets are under examination [11,12,13]. The screening of drugs approved by the FDA for other purposes is an attractive strategy [14], as this has the potential of providing expedited treatment of SARS-CoV-2 infection [9,15,16]. Specifically, targeting the ACE2-RBD assembly with low molecular weight drugs [17,18] and identifying other small molecules interfering with this recognition mechanism will prevent cell invasion by the virus, and thereby block infection [19].

Antimalarial drugs were among the first to be selected for repurposing as drugs against SARS-CoV-2 [20], for which different mechanisms, such as the inhibition of endocytic pathways by elevation of endosomal pH and the interference of ACE2 with glycosylation have been proposed [21]. In this respect, activities of chloroquine and other antimalarial drugs such as artemisinins have been reported [22,23,24], although the efficacy of chloroquine is debatable [25]. In the current study, the interaction of natural and synthetic compounds with antimalarial activity with the RBD and ACE2 are examined.

## 2. Results and Discussion

### 2.1. Antimalarial Compounds against SARS-CoV-2

**Figure 1:**
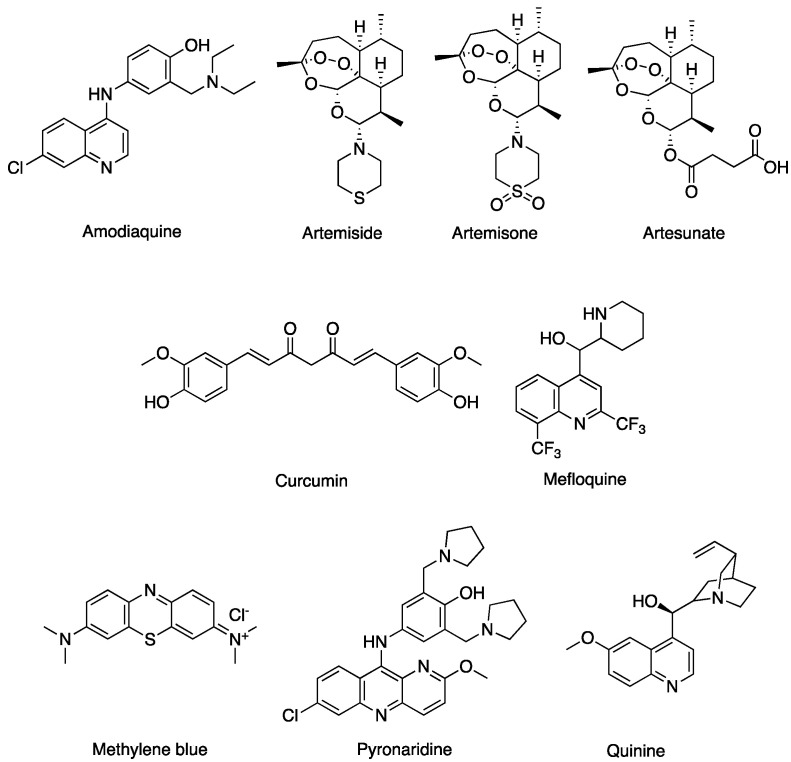
Structures of the antimalarial compounds used in this investigation.

The drugs selected for examination included artemisinins, quinine and analogues and other compounds known to be active against malaria (Figure 1). Amodiaquine is active against other coronaviruses [26] and, in combination with nelfinavir, is active against SARS-CoV-2 [27]. Artemisinins including artesunate and the newer amino-artemisinins, artemiside and artemisone [28], are prepared by synthetic modification of the parent drug, artemisinin, obtained from the sweet wormwood *Artemisia annua* that has been used in Chinese traditional medicine for treating fevers. Artemisinins are shown to reduce the production of SARS-CoV-2 proteins, block infection at post entry level and interfere with the RBD [22,29]. More recently, artesunate has been selected for a Phase III study in COVID-19 patients, essentially based on its anti-inflammatory, rather than virucidal, properties (WHO Solidarity Trial, https://www.isrctn.com/ISRCTN18066414, accessed on 15 September 2021). Curcumin is a well-known bioactive natural product that acts against different targets [30,31,32,33]. According to virtual screening, it has the potential of targeting the ACE2-RBD assembly [34]. Methylene blue is approved by the FDA for the treatment of methemoglobinemia and is an active antimalarial drug. The compound prevents the interaction of S1 with ACE2 and consequently inhibits viral entry [35]. Mefloquine, quinine and pyronaridine display antiviral activity against SARS-CoV-2 in vitro [21,26]. The activity of pyronaridine against SARS-CoV-2 has also been evaluated by measuring its interaction with the RBD by microscale thermophoresis [36,37].

### 2.2. Docking Studies

**Figure 2:**
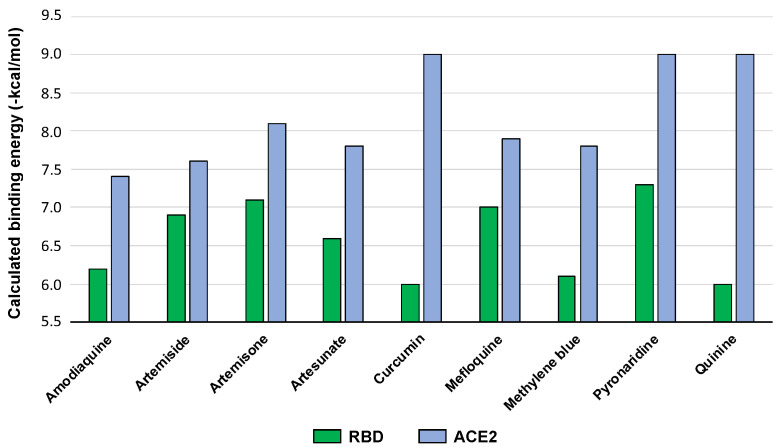
Calculated binding energies for the interaction of the compounds with RBD (PDB ID: 6VSB) and ACE2 (PDB ID: 6LZG).

Virtual screening techniques have been adopted to identify compounds, often among sets of known drugs or natural products to target SARS-CoV-2 or host proteins [38,39,40,41,42]. Ligand-based approaches, docking and molecular dynamics (MD) simulation have been used per se or in combination with in vitro assays [43,44]. In the current study, molecular docking was used to conduct a preliminary screen of the compounds and assess the predicted interaction motifs with the RBD and ACE2. Better calculated binding energies were found for the compounds with ACE2. Pyronaridine appeared to be the most promising molecule, since good binding energies were computed with both the RBD and ACE2. Artemisone also emerged as a promising RBD binder (Figure 2, Table S1 in the Supplementary Material for computed values).

**Figure 3:**
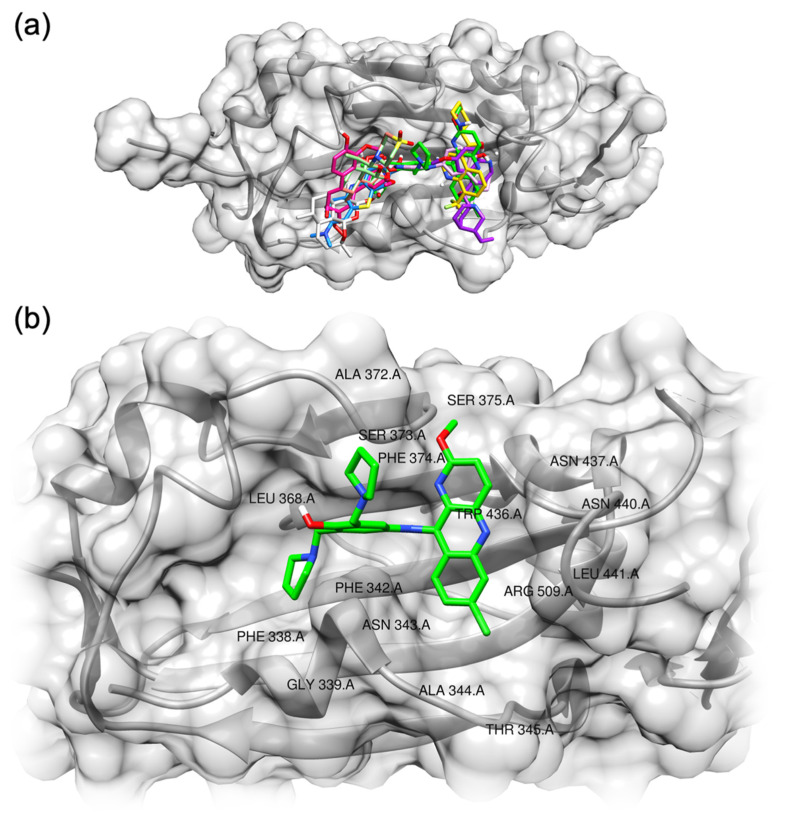
(**a**) Predicted binding motif for the compounds with RBD; (**b**) detailed view of the residues interacting with pyronaridine.

Interestingly, the compounds interact with the same site of the RBD and thus form a single cluster (Figure 3, Figures S1–S9 in the Supplementary Material). This region of the RBD includes the 340–510 residues. According to the docking results, pyronaridine interacts with some hydrophobic residues (Phe342, Ala344, Ala372, Phe374 and Leu441) and also with several polar amino acids (Asn343, Thr345, Ser373, Ser375, Trp436, Asn437, Asn440 and Arg509, Figure 3b and Figure S8 in the Supplementary Material).

**Figure 4:**
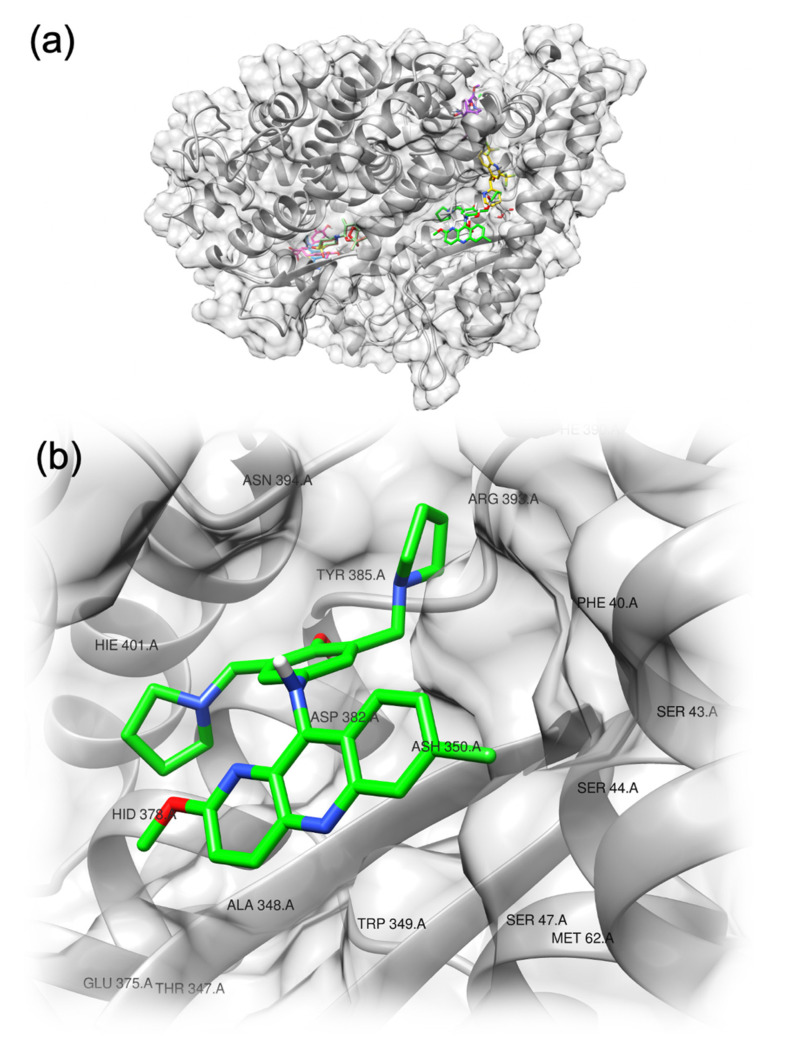
Predicted binding motif for the compounds with ACE2 (**a**). Detailed view of the residues interacting with pyronaridine (**b**).

For ACE2, the docking experiment indicated more than one interaction site (Figure 4). Artemiside and artemisone share a similar interaction motif as they target the same residues within the pocket (Tyr127, Leu144, Glu145, Asn149, Leu503, Phe504, His505). Similarly, curcumin and methylene blue share a common binding pattern, but at a different site (Asn290, Ale413, Thr414, Glu435, Phe438). Artesunate, mefloquine, quinine and pyronaridine interact with adjacent pockets (residues 340–400), while amodiaquine interacts with the same region of the protein but through a different pool of residues (Gln98, Ala99, Gln102, residues 205-209, Ala396, Asn397) (Figures S10–S18 in the Supplementary Materials). Again, pyronaridine was the best binder of the set, together with curcumin and quinine. Interestingly, for pyronaridine, the binding site on ACE2 is characterized by the presence of several polar residues, as was also observed in the case of binding to the RBD.

### 2.3. Molecular Dynamics Simulations

**Figure 5:**
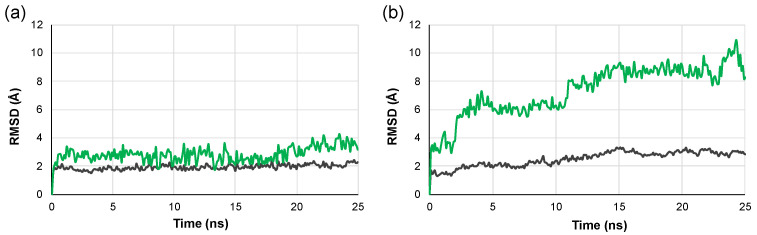
MD simulations for the complexes of pyronaridine with (**a**) RBD and (**b**) ACE2 obtained from docking experiments. Root mean square deviation (RMSD) is depicted in green for the ligand and in dark grey for the protein.

As docking studies indicated that pyronaridine was the most promising RBD and ACE2 binder according to its docking scores, preliminary MD simulations were carried out to assess the stability of the computed pyronaridine ligand-target complex. The experiments showed that the pyronaridine-RBD complex is stable, and that the ligand is retained in the binding site within the simulation time. On the other hand, the interaction of pyronaridine with ACE2 is characterized by limited stability, especially if the trajectory of the ligand is considered (Figure 5).

### 2.4. Bio-Layer Interferometry

The binding of the compounds was then assayed in vitro using bio-layer interferometry, a label-free technology for measuring biomolecular interactions [45,46,47]. This experiment is based on the immobilization of the target protein on the biosensor, which is followed by its exposure to different ligand concentrations, allowing for the real-time measurement of association and dissociation events. Bio-layer interferometry allows the calculation of the association rate (k_on_), dissociation rate (k_dis_) and dissociation constant (K_D_). This technique has been adopted to screen RBD ligands [17,48] in combination with virtual screening [49,50]. The results of the bio-layer experiments are presented in Table 1. All the compounds bind to ACE2 with overall good affinity values (10^−6^ < K_D_ < 10^−4^, see Figures S27–S34 in the Supplementary Material). These results confirm the observations from the docking studies where the compounds showed good, calculated binding energy values against ACE2 (<−7.5 kcal/mol, except for amodiaquine). However, the compounds showed different behavior towards the RBD. No interaction with the RBD was able to be measured for artemiside, artesunate, curcumin and quinine. In contrast, artemisone, methylene blue and pyronaridine were strong RBD binders (10^−7^ < K_D_ < 10^−5^), showing good correlation (R^2^) values (Figures S19–S26 in the Supplementary Material). Further, artemisone and methylene blue bind in a dose-dependent manner; the binding curves suggested 1:1 binding with R^2^ values of 0.9381 and 0.9864, respectively. The K_D_ values for the interaction of the two compounds were 0.36 and 0.22 μM, respectively. Moreover, pyronaridine binds the RBD with a K_D_ value of 57 μM (R^2^ = 0.9236). As anticipated, higher calculated binding energy values were obtained in docking experiments with the RBD than with ACE2.

**Figure 6:**
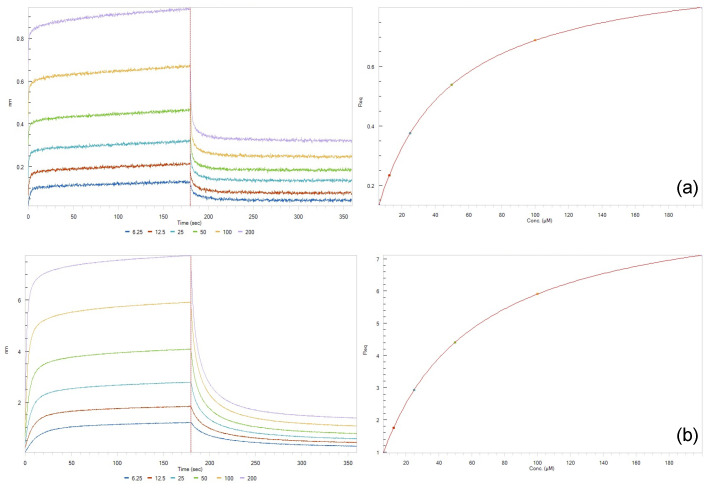
Binding kinetics and affinity analysis for the interaction of pyronaridine with RBD (**a**) and ACE2 (**b**) according to bio-layer interferometry studies.

Taken together, these data suggested that some of the compounds bind with both the viral RBD and host ACE2 receptor. They, thus, may interfere with the recognition process and thereby exhibit antiviral effects. The in vitro studies confirmed the results of the in silico experiments, which indicated that pyronaridine binds to both the RBD and ACE2 (Figure 6) and that artemisone is a good binder for the RBD.

### 2.5. Enzyme-Linked Immunosorbent Assay

**Figure 7:**
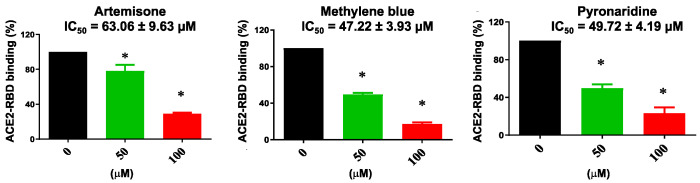
Results of ELISA experiments demonstrating that pyronaridine, artemisone and methylene blue inhibit the binding of RBD to ACE2 (* *p* < 0.05, compared with control group).

The activities of the compounds showing the highest affinity for the RBD were investigated using an ELISA-based test involving immobilized ACE2-His-tag protein on nickel-coated microplates [51,52,53]. This experiment enables the study of the effect of a ligand on the assembly generated by the interaction of RBD with ACE2. Thus, the inhibition (%) of the interaction between the two proteins according to the variation in the optical density of treated wells compared to that of control wells was calculated. As depicted in Figure 7, artemisone, methylene blue and pyronaridine inhibited the binding of RBD to ACE2 in a dose-dependent manner, suggesting that they could inhibit the interaction between the viral RBD peptide and the ACE2 receptor of the host cell. In particular, the *IC*_50_ values of 63.06 ± 9.63, 47.22 ± 3.93 and 49.72 ± 4.19 µM for artemisone, methylene blue and pyronaridine indicate the potential inhibitory effects on the fusion between the viral RBD and the ACE2 receptor of the host cell.

## 3. Materials and Methods

### 3.1. Docking Studies

Structures of the protein targets were obtained from the RCSB Protein Data Bank (www.rcsb.org, accessed on 19 September 2021, PDB IDs: 6VSB, 6LZG), in accordance with previous studies [54,55,56,57]. Protein structures were processed in order to remove ligands and water molecules, while hydrogen atoms were added using standard geometries. Ligands were also prepared for the blind docking experiments, which were performed using Autodock Vina (Molecular Graphics Laboratory, Department of Integrative Structural and Computational Biology, The Scripps Research Institute, La Jolla, CA, USA) [58]. Default docking parameters were selected, setting the search volume grid to encompass the whole protein in the blind docking experiments. Advanced Vina options were set as in the following: number of binding modes, 9; exhaustiveness of search, 8; maximum energy difference (kcal/mol), 3. Output data (energies, interaction patterns) were analyzed and scored using UCSF Chimera molecular viewer [59], which was also used to produce the artwork. Values are expressed in kcal/mol and refer to the most favored predicted pose.

### 3.2. Molecular Dynamics Simulations

MD simulations were carried out using PlayMolecule (Accelera, Middlesex, UK) starting from the output models of docking experiments. The ligand was prepared by running Parametrize function based on GAFF2 force field [60]. The complex was prepared for the simulation using ProteinPrepare and SystemBuilder functions, setting pH = 7.4, AMBER force field and default experiment parameters [61]. Simulations of 25 ns were carried out using SimpleRun, with default settings [62]. Plotting of root mean square deviation (RMSD) was performed using Excel 15.31 (Microsoft, Redmond, WA, USA).

### 3.3. Chemicals

Pyronaridine, amodiaquine and mefloquine were provided by Dr. Paolo Saul Coghi. Artemisone and artemiside were provided by Prof. Richard Haynes. Other reagents and solvents (unless stated otherwise) were purchased from commercial sources (Aurora Borealis, Taipei City, Taiwan) and used without further purification.

### 3.4. Bio-Layer Interferometry

Compounds were diluted to the appropriate concentrations (1–100 μM) with PBS. Purified SARS-CoV-2 RBD peptide (Sino Biological, Beijing, China) was conjugated with biotin using EZ-LinkTM Sulfo-NHS-Biotin (Genemore, Beijing, China) following the protocol reported by the manufacturer. The biotinylated SARS-CoV-2 RBD peptide was immobilized onto Super Streptavidin (SSA) biosensors (Fortebio, Fremont, CA, USA). ACE2-His (Sino Biological, China) was immobilized onto the biosensor coated with nickel-nitrilotriacetic acid (Ni-NTA, Fortebio, USA). After washing (60 s) and baseline step with PBS containing 2% DMSO (Merck, Darmstadt, Germany), the biosensor tips were immersed into the wells containing serial dilutions and allowed to associate (300 s). A dissociation step was then performed (300 s). K_D_ values were calculated using a 1:1 binding model in Data Analysis Software 9.0 (Fortebio, CA, USA), using k_dis_ as the dissociation constant (s^−1^) and k_on_ as the association constant (1/Ms). K_D_ is computed from k_dis_/k_on_.

### 3.5. Enzyme-Linked Immunosorbent Assay

The SARS-CoV-2 Spike-ACE2 Inhibitor Screening Assay Kit (Cat: #79931) was purchased from BPS Biosciences (San Diego, CA, USA). Nickel-coated 96-well white microplates were coated with 1 μg/mL ACE2-His-tag protein following the protocol of the manufacturer. Briefly, 1 ng/uL SARS-CoV-2 RBD was added to ACE2-His-tag coated test wells in the presence of 0, 50 or 100 μM solution of tested compound. Wells without the compounds and SARS-CoV-2 RBD protein were set as blank control. ELISA ECL substrate solution containing anti-His horseradish peroxidase was added into the microplate wells followed by the chemiluminescence measurement using the luminometer (Tecan, Männedorf, Switzerland). The data were then analyzed using Prism 7.0 (GraphPad, San Diego, CA, USA) and *IC*_50_ was calculated according to the following equation:

(1) $$Y=Bottom+\frac{Top-Bottom}{1+\frac{X}{IC_{50}}}$$

Concentration of tested compound is reported on the *X* and response on the *Y* axes. “*Top*” and “*Bottom*” indicate plateaus in the units of the *Y* axis, representing maximum and minimum measured activity values. Quantification bars were obtained from the results of 3 independent experiments.

## 4. Conclusions

Bio-layer interferometry studies indicated that artemisone, methylene blue and pyronaridine are strong RBD binders. Most of the compounds also demonstrated a good binding affinity for ACE2. The use of MD simulations demonstrated that the compounds form stable complexes with the RBD. Indeed, higher affinity for the RBD is a desirable feature for antiviral agents, as avoiding binding with ACE2, which is a widespread human receptor, will prevent undesired side effects. Artemisone and methylene blue showed even higher affinity for the RBD and higher selectivity over ACE2.

Whilst the bio-layer interferometry results indicate the compounds may interact with the isolated target proteins, the ELISA experiments confirmed that artemisone, methylene blue and pyronaridine effectively counteract the interaction between the RBD and ACE2-RBD; thus, they may prevent cell invasion by SARS-CoV-2 through this pathway.

Molecular docking identified artemisone and pyronaridine as ligands for the targets (−7.1 and −7.3 kcal/mol for RBD and −8.1 and −9.0 kcal/mol for ACE2, respectively), as confirmed in vitro by the bio-layer and ELISA experiments. Preliminary MD studies demonstrated the stability of the ligand–RBD complex. However, as the biological effects were observed at relatively high concentrations for some of the molecules, the optimization of compound structures will be required. Overall, these results support the reliability of the screening protocols described here and paves the way for the identification of more efficient and selective ligands.

## Figures and Tables

**Table 1:** Results of bio-layer interferometry experiments for probing the interaction of the compounds with the RBD and ACE2 in vitro. Calculated association rate (k_on_, 1/Ms), dissociation rate (k_dis_, s^−1^) and dissociation constant (K_D_, M) are reported.

	RBD				ACE2			
Compound	K_D_ (M)	k_on_	k_dis_	R^2^	K_D_ (M)	k_on_	k_dis_	R^2^
Amodiaquine	9.87 × 10^−6^	2.34 × 10^5^	2.31	0.7332	3.13 × 10^−5^	2.10 × 10^3^	6.57 × 10^−2^	0.9735
Artemiside	-	-	-	-	1.24 × 10^−4^	4.29 × 10^3^	5.33 × 10^−1^	0.9463
Artemisone	3.63 × 10^−7^	8.40 × 10^7^	3.05 × 10	0.9381	6.81 × 10^−4^	9.71 × 10^2^	6.61 × 10^−1^	0.9235
Artesunate	-	-	-	-	7.60 × 10^−5^	1.29 × 10^3^	9.07 × 10^−2^	0.6905
Curcumin	-	-	-	-	2.03 × 10^−5^	6.00 × 10^2^	1.22 × 10^−2^	0.8548
Mefloquine	6.05 × 10^−3^	1.42 × 10^2^	8.61 × 10^−1^	0.9109	4.84 × 10^−4^	7.16 × 10^2^	3.12 × 10^−2^	0.9826
Methylene blue	2.26 × 10^−7^	5.08 × 10^6^	1.15	0.9864	4.75 × 10^−4^	2.30 × 10^2^	1.09 × 10^−1^	0.9898
Pyronaridine	5.68 × 10^−5^	5.98 × 10^2^	3.39 × 10^−2^	0.9236	5.13 × 10^−5^	5.84 × 10^2^	2.99 × 10^−2^	0.9230
Quinine	-	-	-	-	9.35 × 10^−6^	8.77 × 10^2^	8.19 × 10^−3^	0.6316

## Data Availability

The data presented in this study are available on request from the corresponding author.

## Supplementary Materials

The following are available online at https://www.mdpi.com/article/10.3390/ph14100954/s1, Table S1: calculated binding energy values for the interaction between compounds and the target proteins, Figures S1–S18: detailed view of the predicted interaction motifs of compounds with RBD and ACE2, Figures S19–S34: bio-layer interferometry study for the interaction of the compounds with target proteins.
