# Supplementary material for: A Drug Repurposing Approach for Antimalarials Interfering with SARS-CoV-2 Spike Protein Receptor Binding Domain (RBD) and Human Angiotensin-Converting Enzyme 2 (ACE2)
Source: Pharmaceuticals (Basel). 2021 Sep 23;14(10):954. doi: 10.3390/ph14100954 (PMC8537658; doi:10.3390/ph14100954)
Supplement: Supplementary file 1 [file pharmaceuticals-14-00954-s001.zip › pharmaceuticals-1367701-supplementary.pdf]

Supplementary Material

Molecular modeling

Table S1. Calculated binding energy values for the computed interaction between studied compounds and the target proteins.

	RBD (−kcal/mol)	ACE2 (−kcal/mol)
Amodiaquine	6.2	7.4
Artemiside	6.9	7.6
Artemisone	7.1	8.1
Artesunate	6.6	7.8
Curcumin	6.0	9.0
Mefloquine	7.0	7.9
Methylene blue	6.1	7.8
Pyronaridine	7.3	9.0
Quinine	6.0	9.0

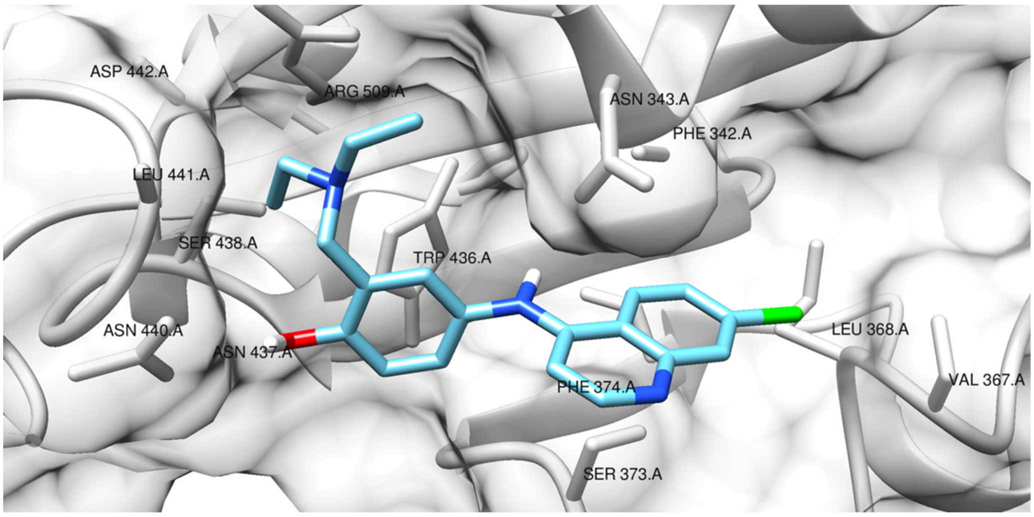

Figure S1. Detailed view of the predicted interaction motif of amodiaquine with RBD.

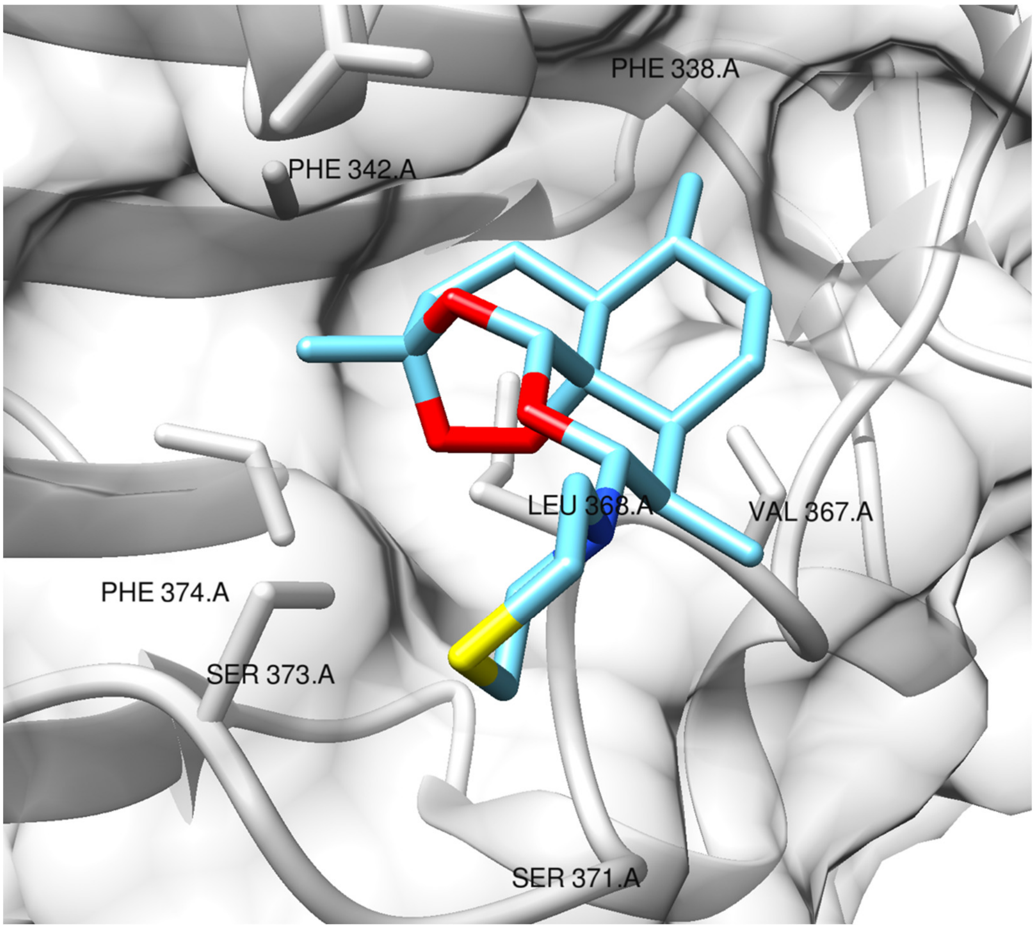

Figure S2. Detailed view of the predicted interaction motif of artemiside with RBD.

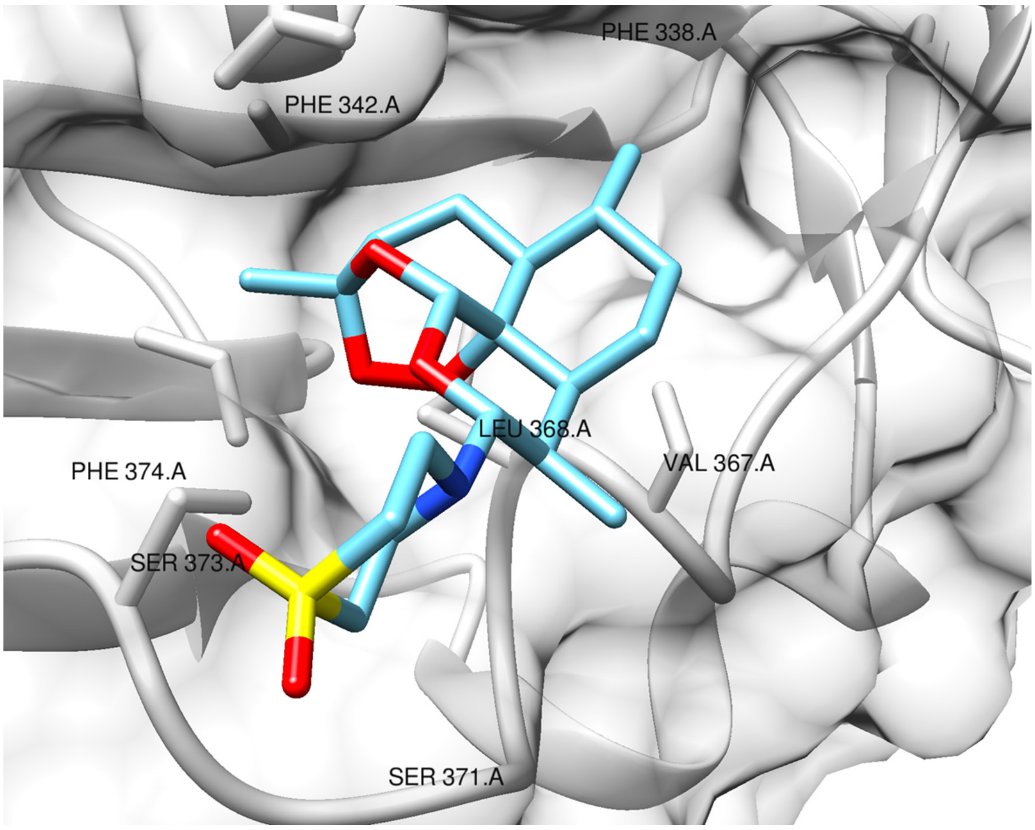

Figure S3. Detailed view of the predicted interaction motif of artemisone with RBD.

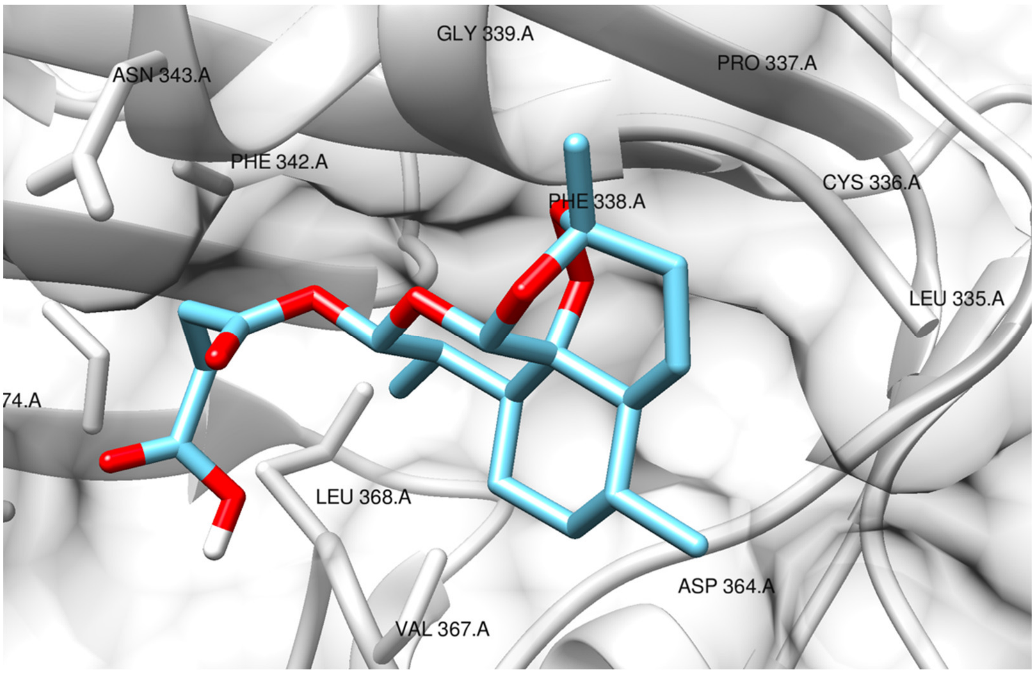

Figure S4. Detailed view of the predicted interaction motif of artesunate with RBD.

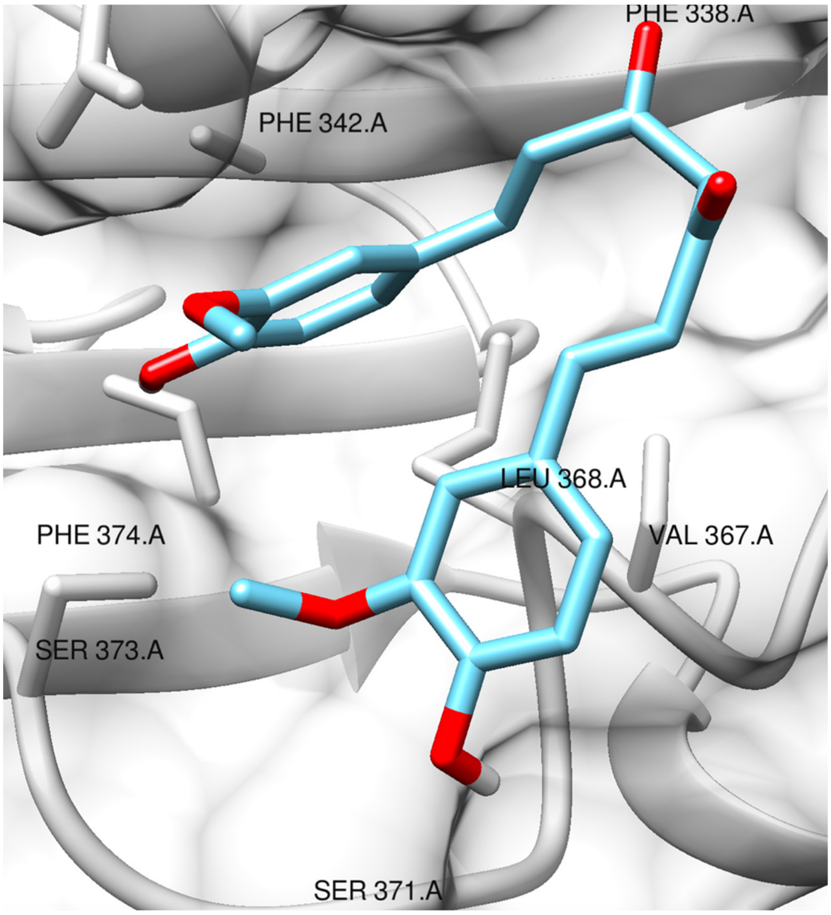

Figure S5. Detailed view of the predicted interaction motif of curcumin with RBD.

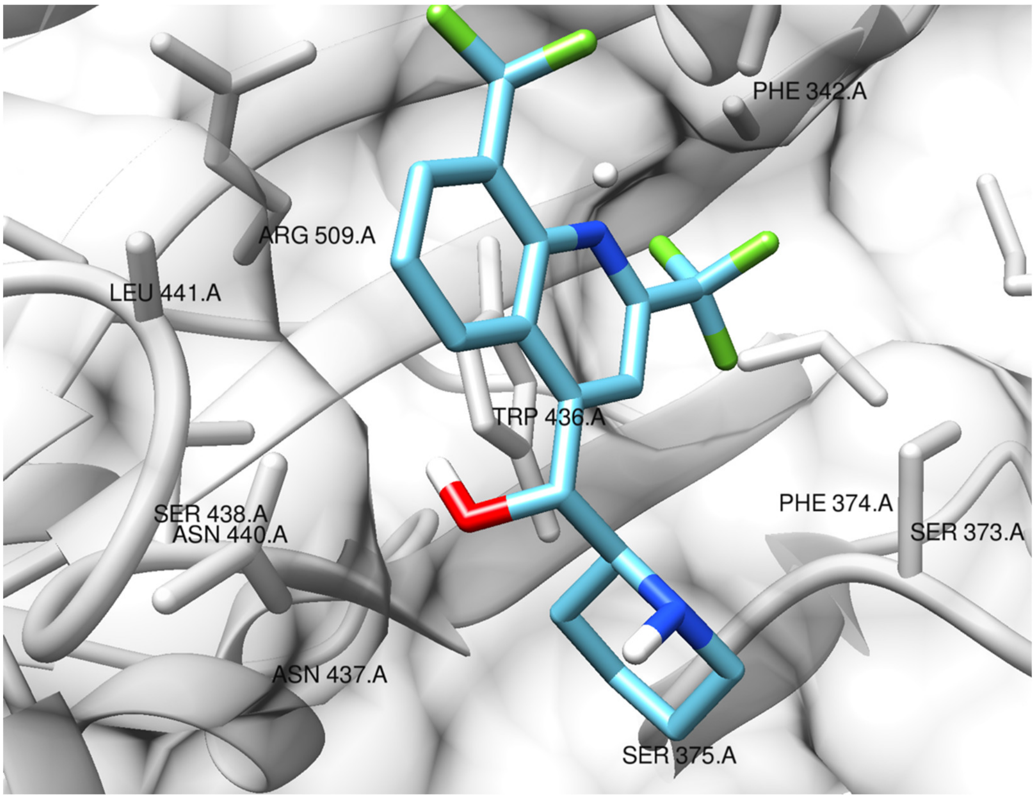

Figure S6. Detailed view of the predicted interaction motif of mefloquine with RBD.

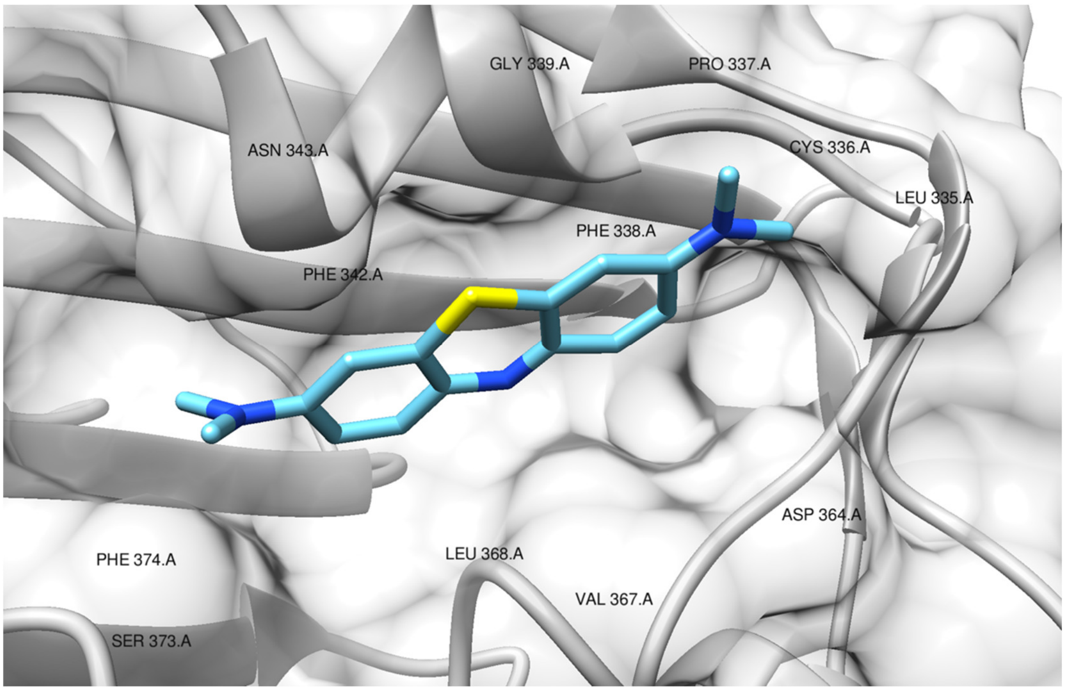

Figure S7. Detailed view of the predicted interaction motif of methylene blue with RBD.

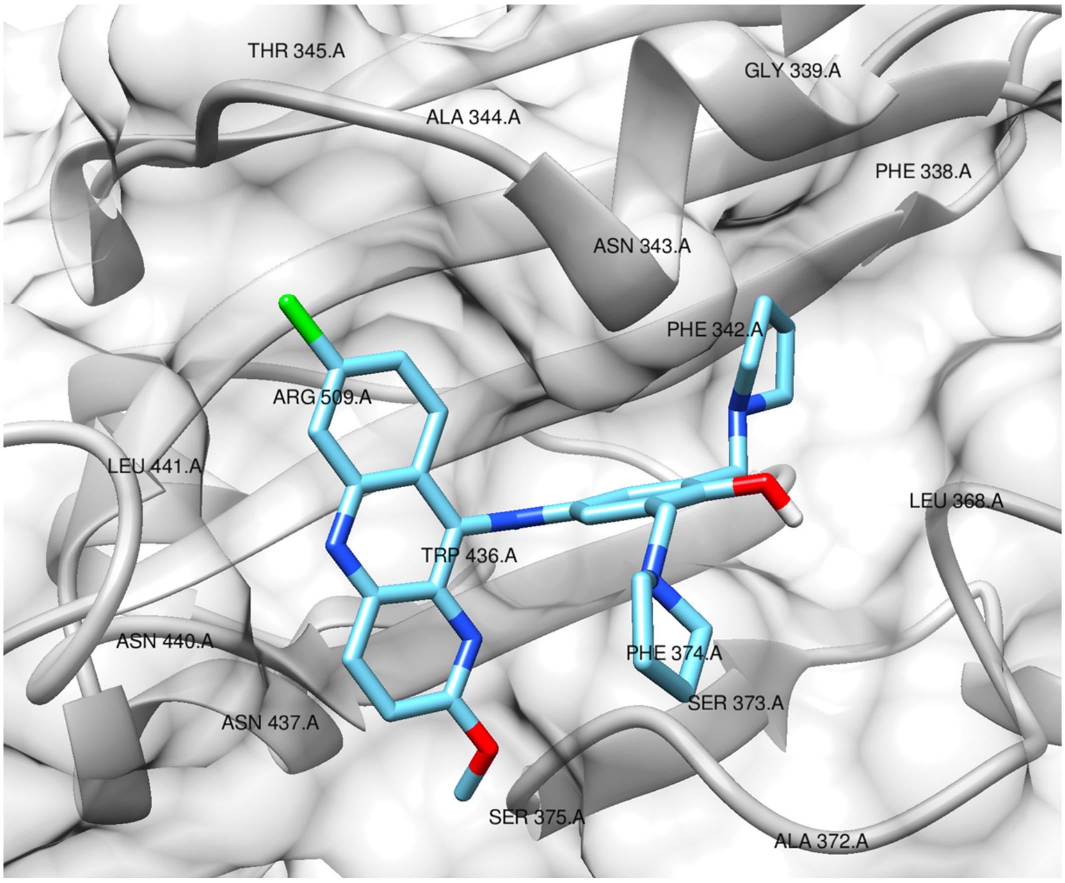

Figure S8. Detailed view of the predicted interaction motif of pyronaridine with RBD.

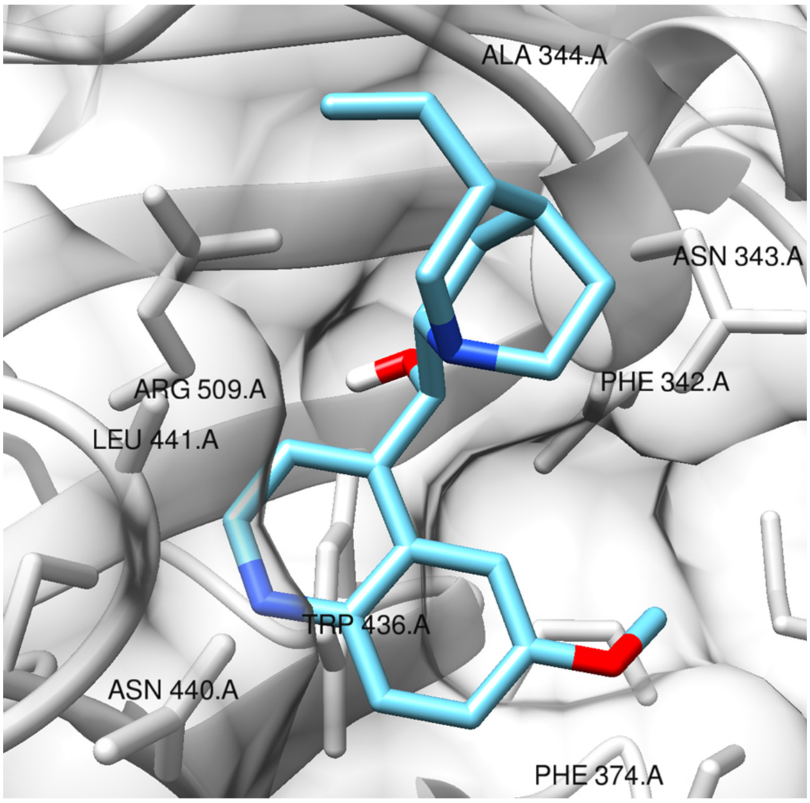

Figure S9. Detailed view of the predicted interaction motif of quinine with RBD.

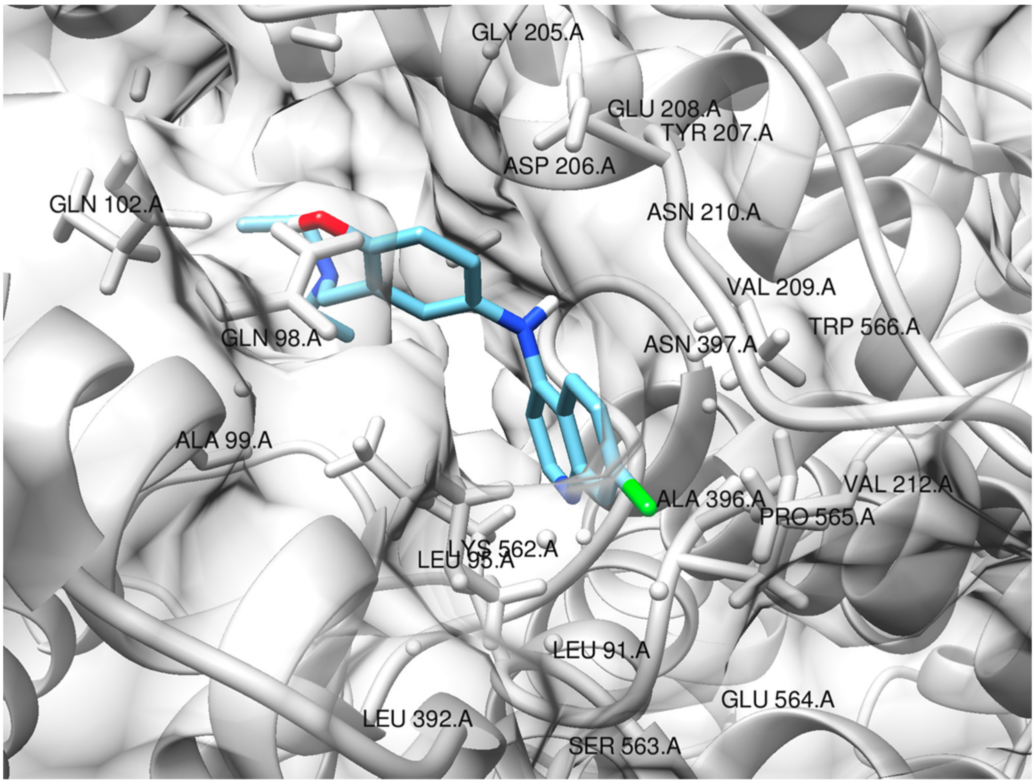

Figure S10. Detailed view of the predicted interaction motif of amodiaquine with ACE2.

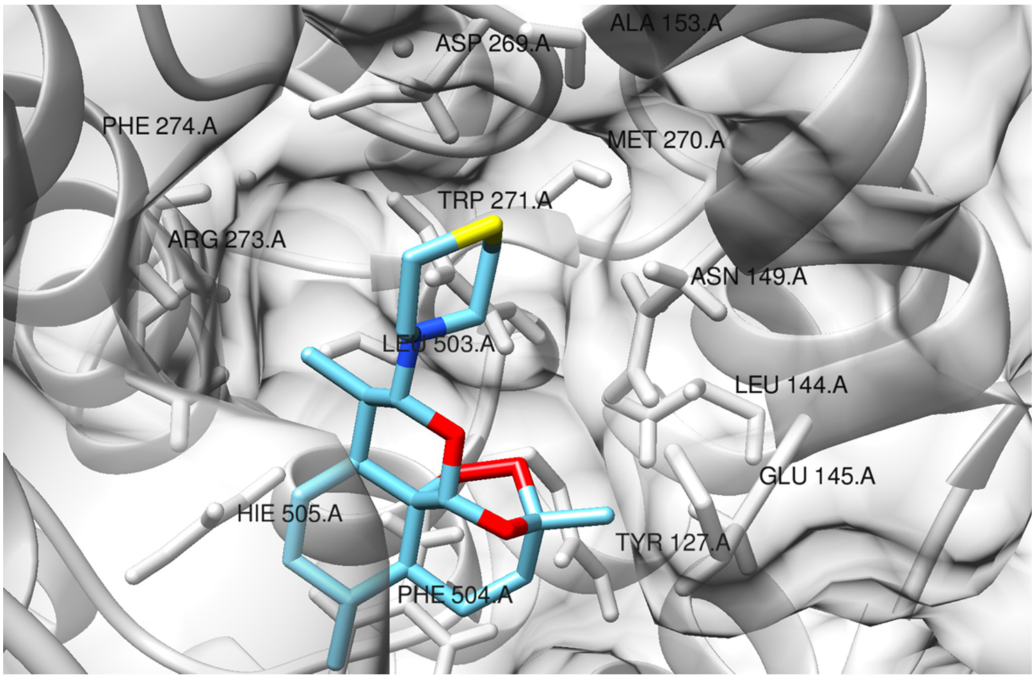

Figure S11. Detailed view of the predicted interaction motif of artemiside with ACE2.

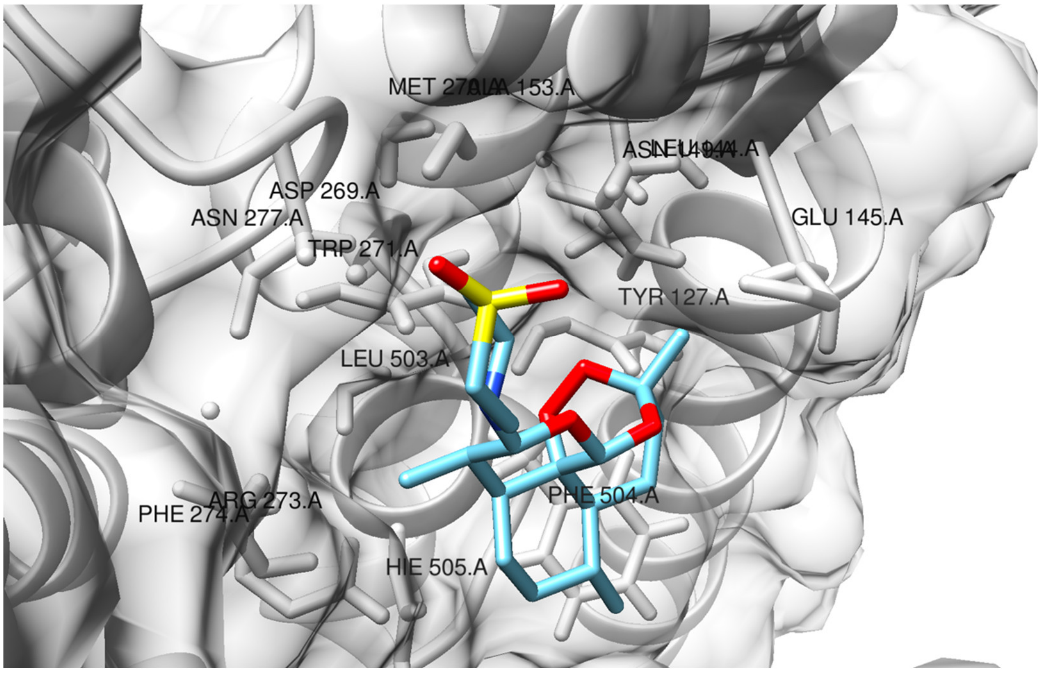

Figure S12. Detailed view of the predicted interaction motif of artemisone with ACE2.

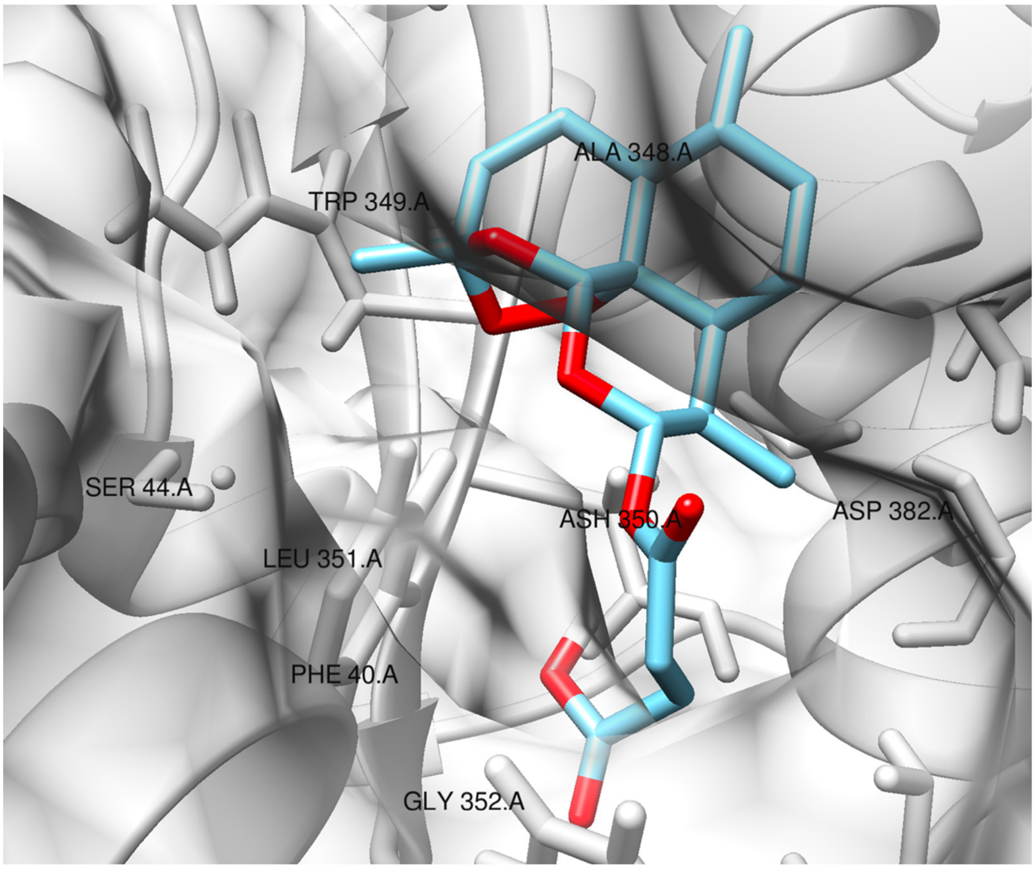

Figure S13. Detailed view of the predicted interaction motif of artesunate with ACE2.

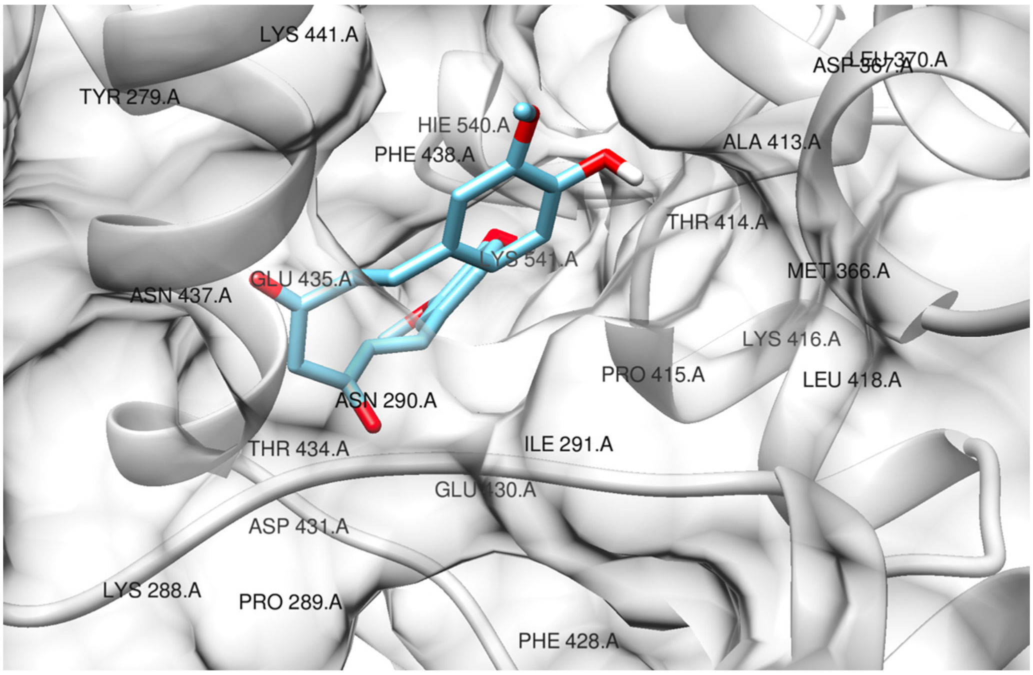

Figure S14. Detailed view of the predicted interaction motif of curcumin with ACE2.

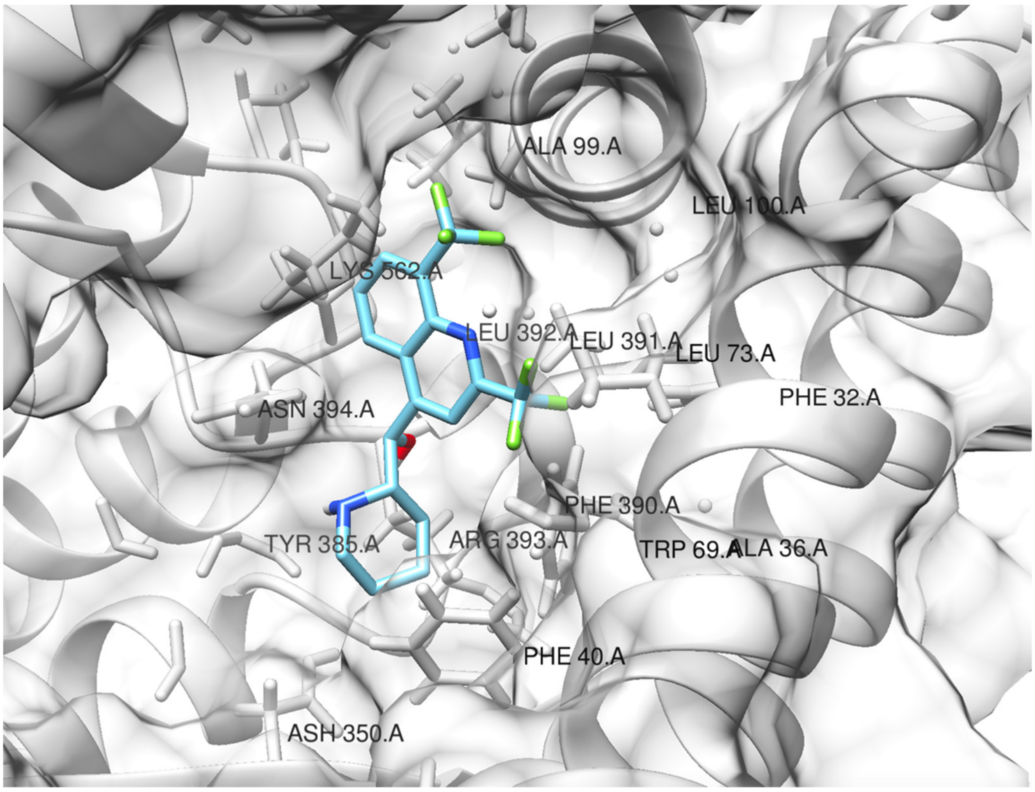

Figure S15. Detailed view of the predicted interaction motif of mefloquine with ACE2.

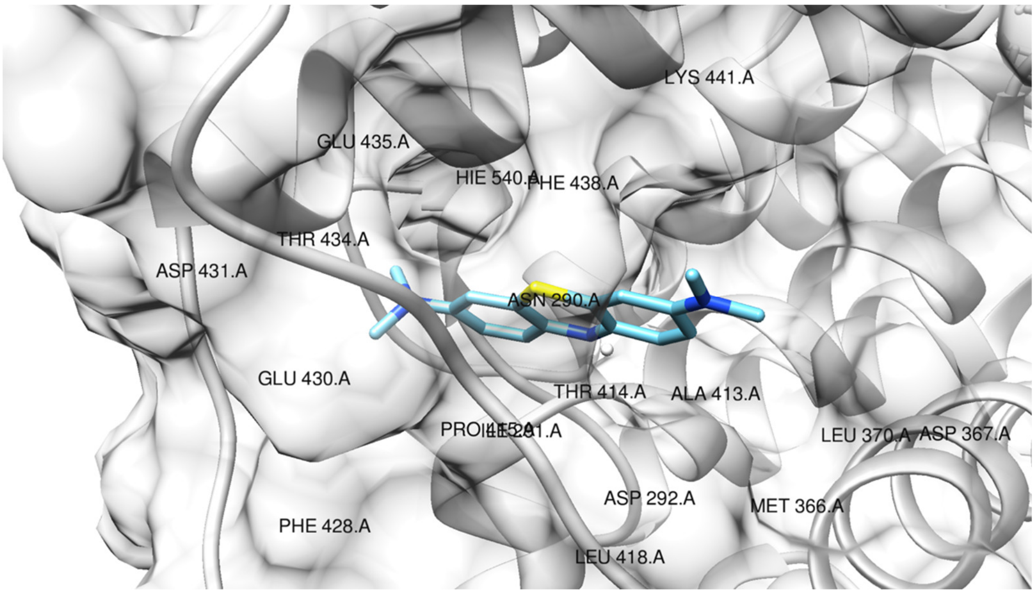

Figure S16. Detailed view of the predicted interaction motif of methylene blue with ACE2.

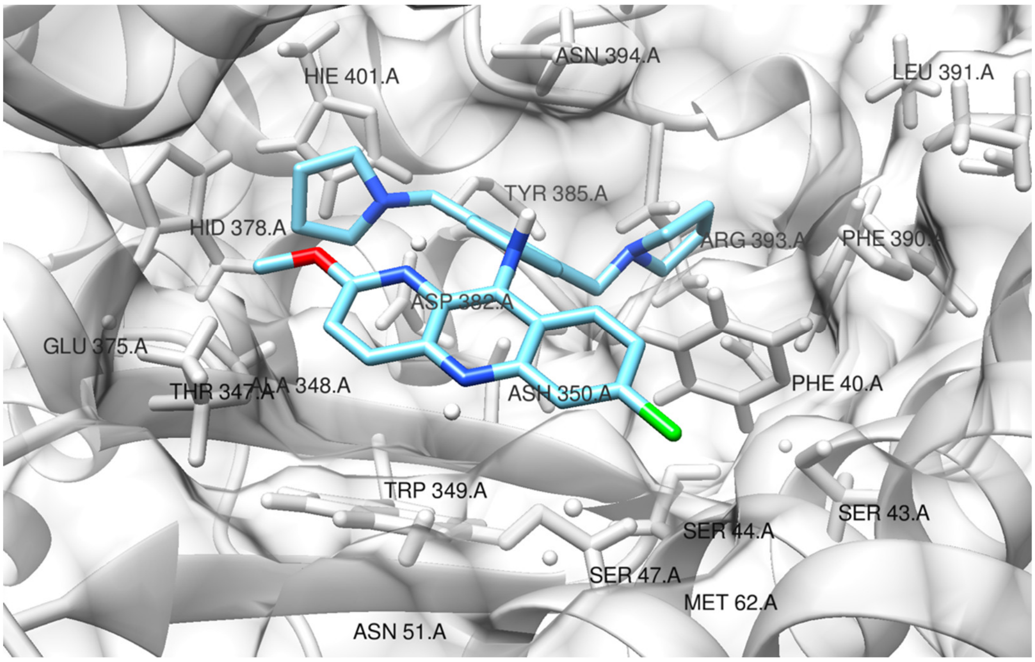

Figure S17. Detailed view of the predicted interaction motif of pyronaridine with ACE2.

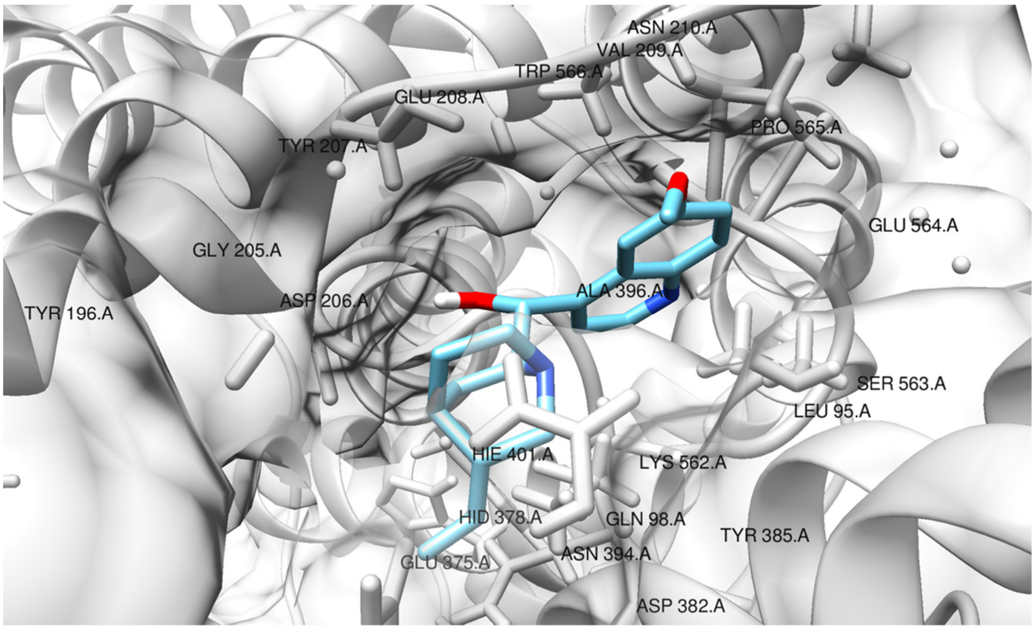

Figure S18. Detailed view of the predicted interaction motif of quinine with ACE2.

Bio-layer interferometry

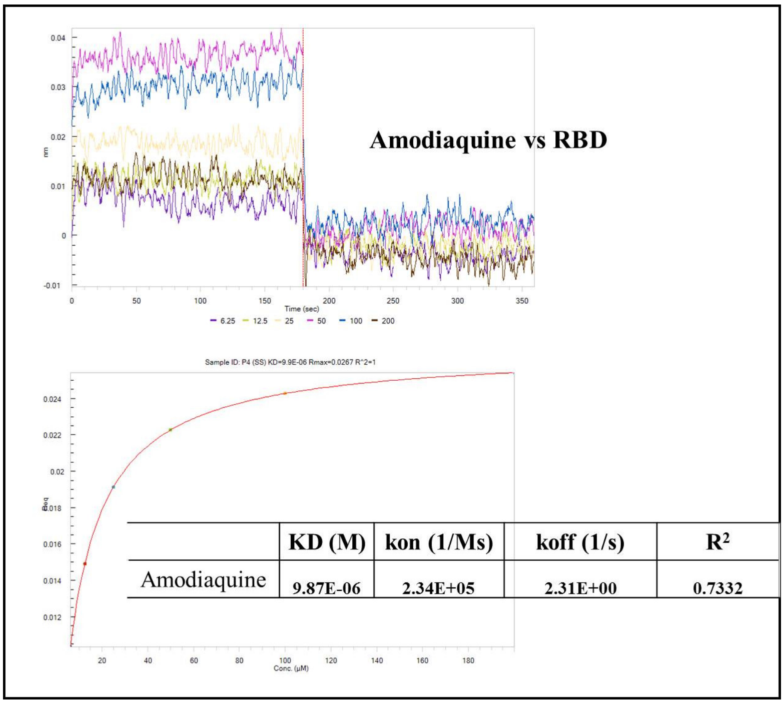

	KD (M)	kon (1/Ms)	koff (1/s)	R^2
Amodiaquine	**9.87E-06**	**2.34E+05**	**2.31E+00**	**0.7332**

Figure S19. Bio-layer interferometry study for the interaction of amodiaquine with RBD.

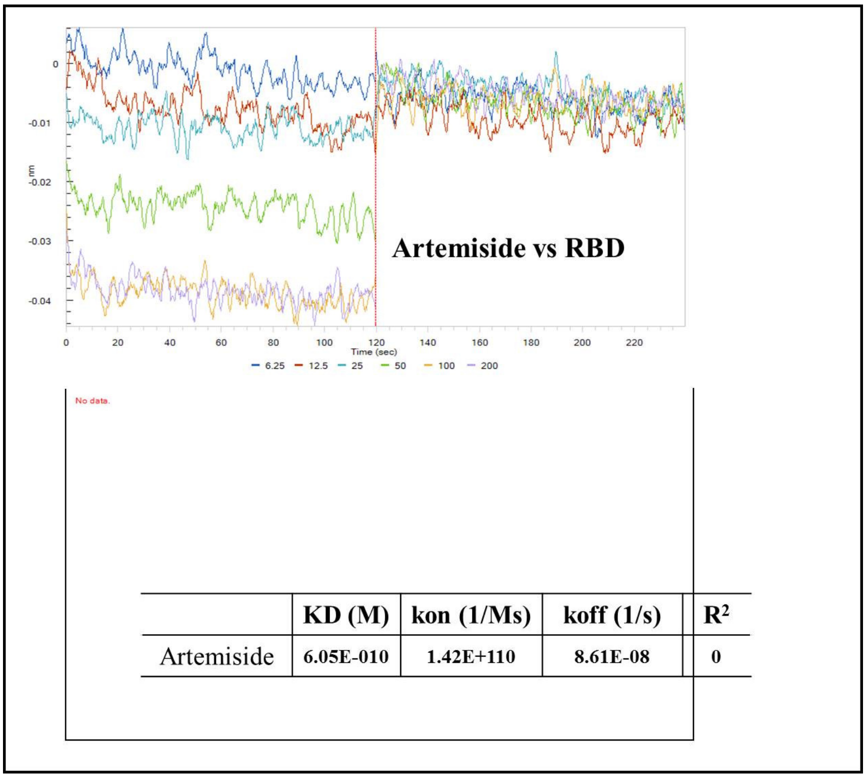

	KD (M)	kon (1/Ms)	koff (1/s)	R^2
Artemiside	**6.05E-010**	**1.42E+110**	**8.61E-08**	**0**

Figure S20. Bio-layer interferometry study for the interaction of artemiside with RBD.

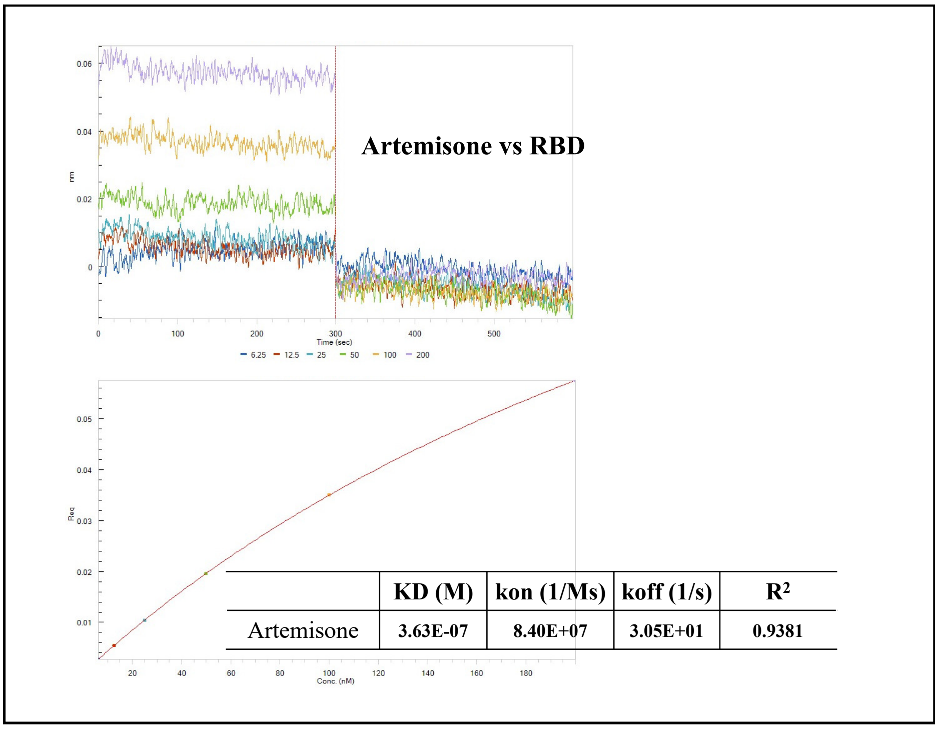

	KD (M)	kon (1/Ms)	koff (1/s)	R^2
Artemisone	**3.63E-07**	**8.40E+07**	**3.05E+01**	**0.9381**

Figure S21. Bio-layer interferometry study for the interaction of artemisone with RBD.

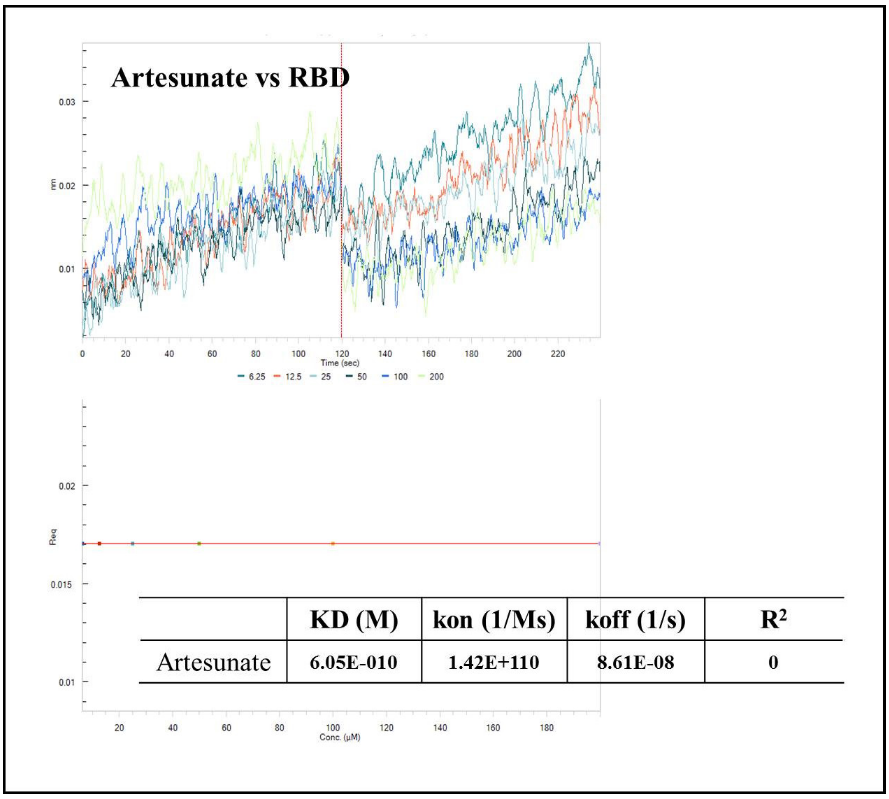

	KD (M)	kon (1/Ms)	koff (1/s)	R^2
Artesunate	**6.05E-010**	**1.42E+110**	**8.61E-08**	**0**

Figure S22. Bio-layer interferometry study for the interaction of artesunate with RBD.

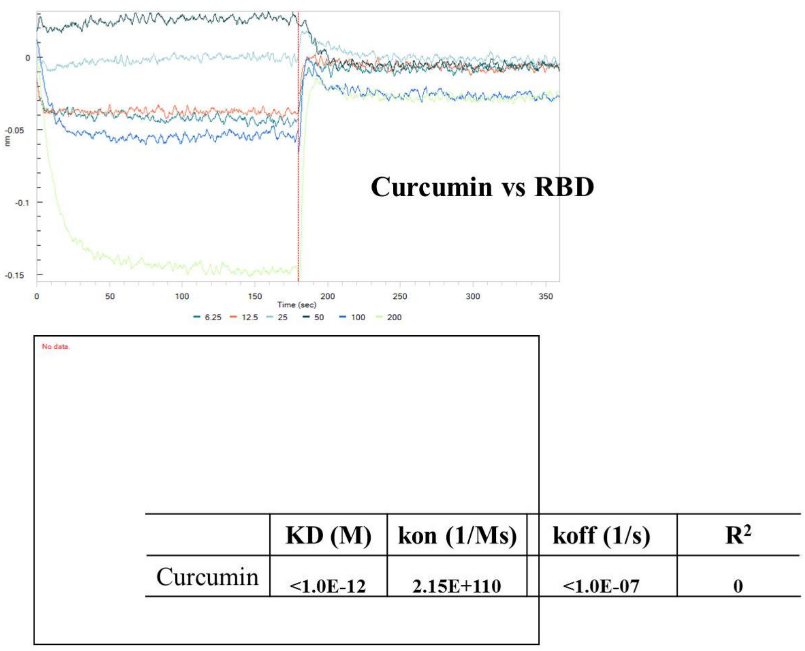

	KD (M)	kon (1/Ms)	koff (1/s)	R^2
Curcumin	<1.0E-12	2.15E+110	<1.0E-07	0

Figure S23. Bio-layer interferometry study for the interaction of curcumin with RBD.

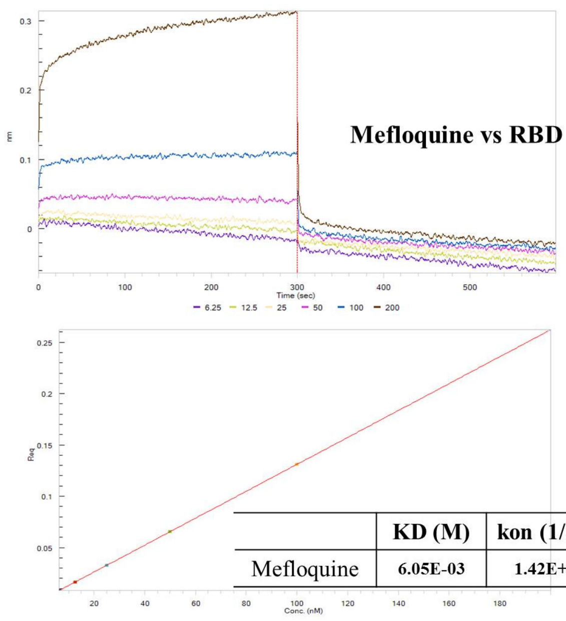

	KD (M)	kon (1/Ms)	koff (1/s)	R^2
Mefloquine	6.05E-03	1.42E+02	8.61E-01	0.9109

Figure S24. Bio-layer interferometry study for the interaction of mefloquine with RBD.

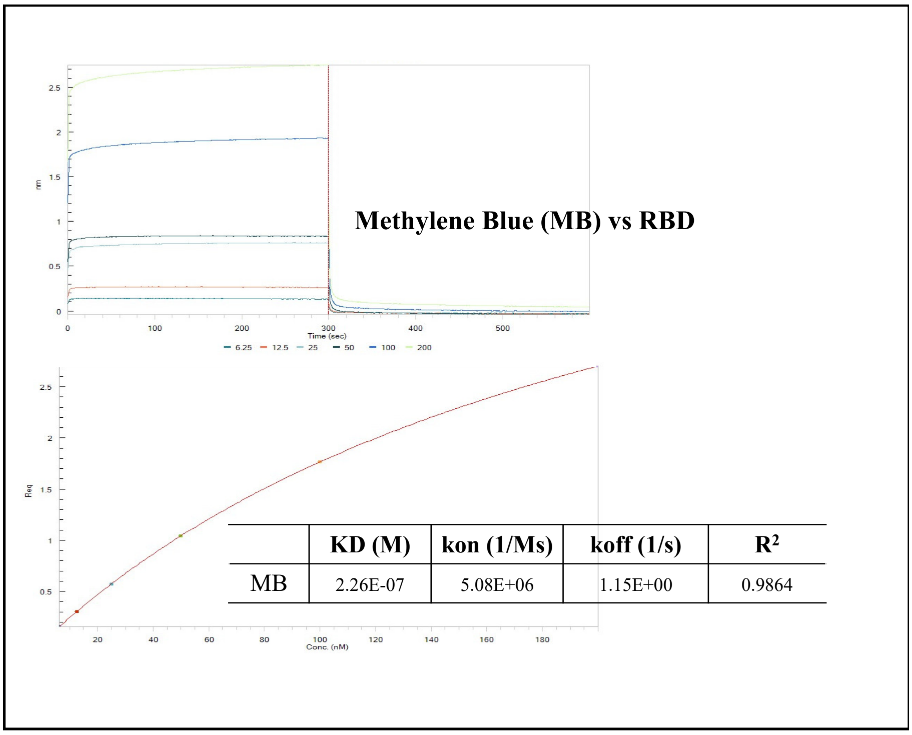

	KD (M)	kon (1/Ms)	koff (1/s)	R^2
MB	2.26E-07	5.08E+06	1.15E+00	0.9864

Figure S25. Bio-layer interferometry study for the interaction of methylene blue with RBD.

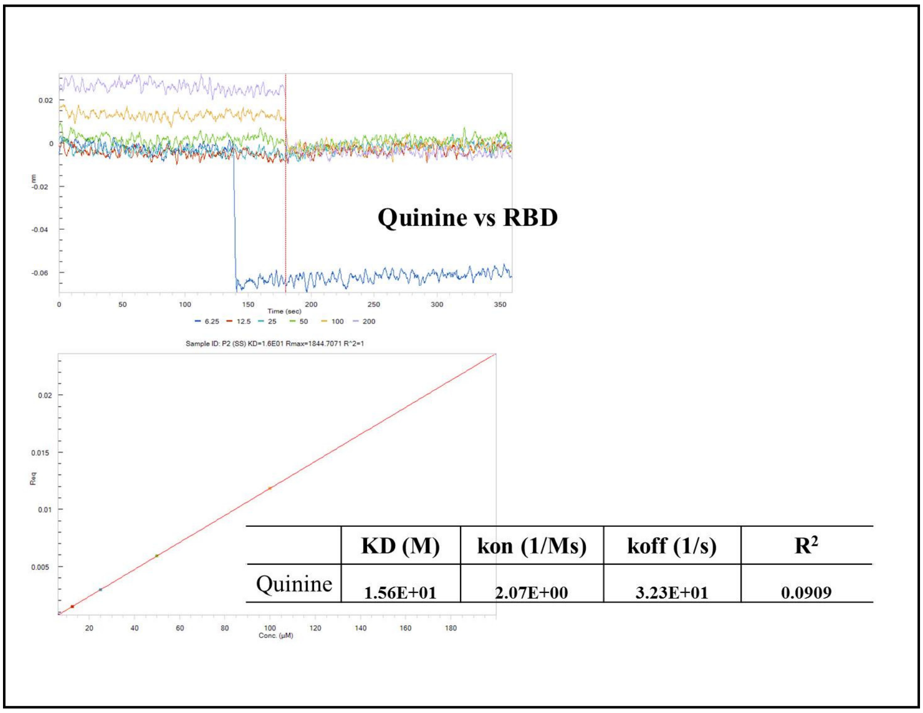

	KD (M)	kon (1/Ms)	koff (1/s)	R^2
Quinine	1.56E+01	2.07E+00	3.23E+01	0.0909

Figure S26. Bio-layer interferometry study for the interaction of quinine with RBD.

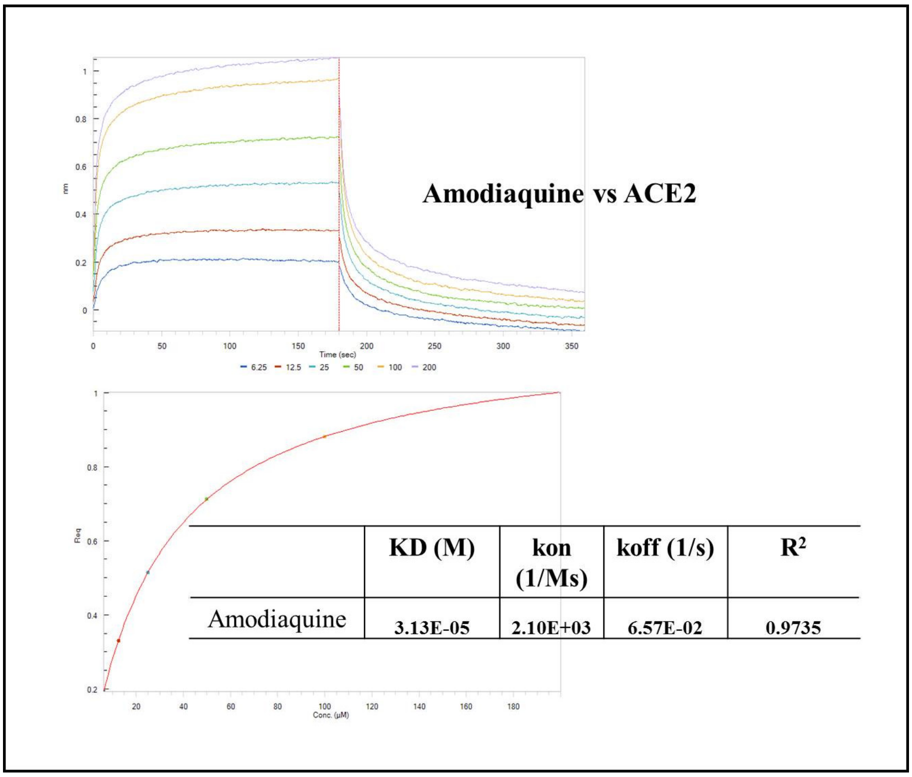

	KD (M)	kon (1/Ms)	koff (1/s)	R^2
Amodiaquine	**3.13E-05**	**2.10E+03**	**6.57E-02**	**0.9735**

Figure S27. Bio-layer interferometry study for the interaction of amodiaquine with ACE2.

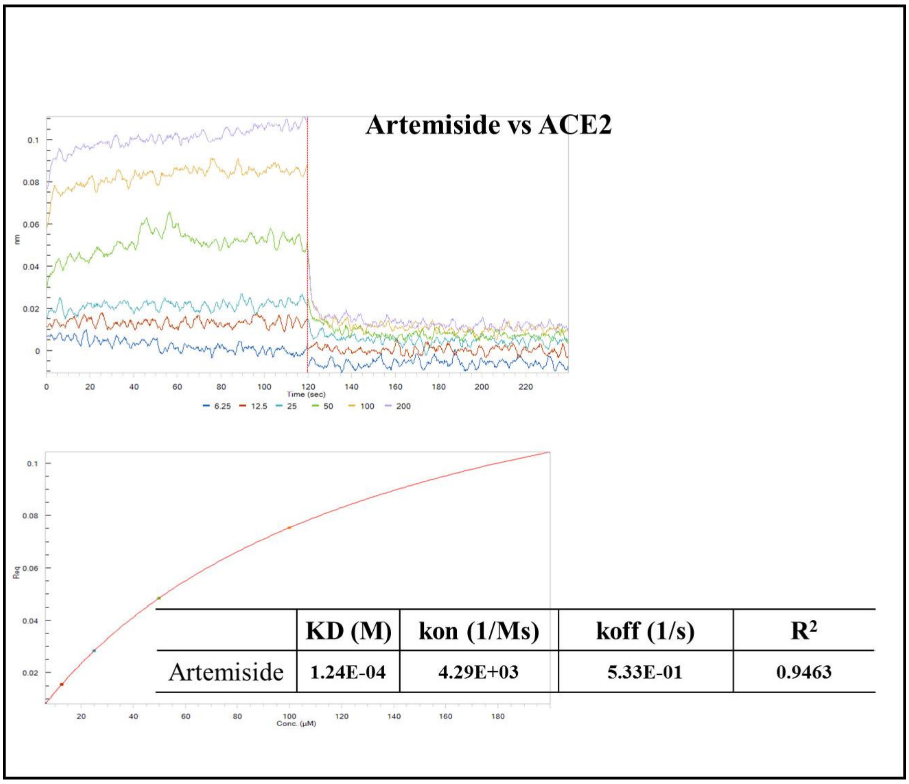

	KD (M)	kon (1/Ms)	koff (1/s)	R^2
Artemiside	**1.24E-04**	**4.29E+03**	**5.33E-01**	**0.9463**

Figure S28. Bio-layer interferometry study for the interaction of artemiside with ACE2.

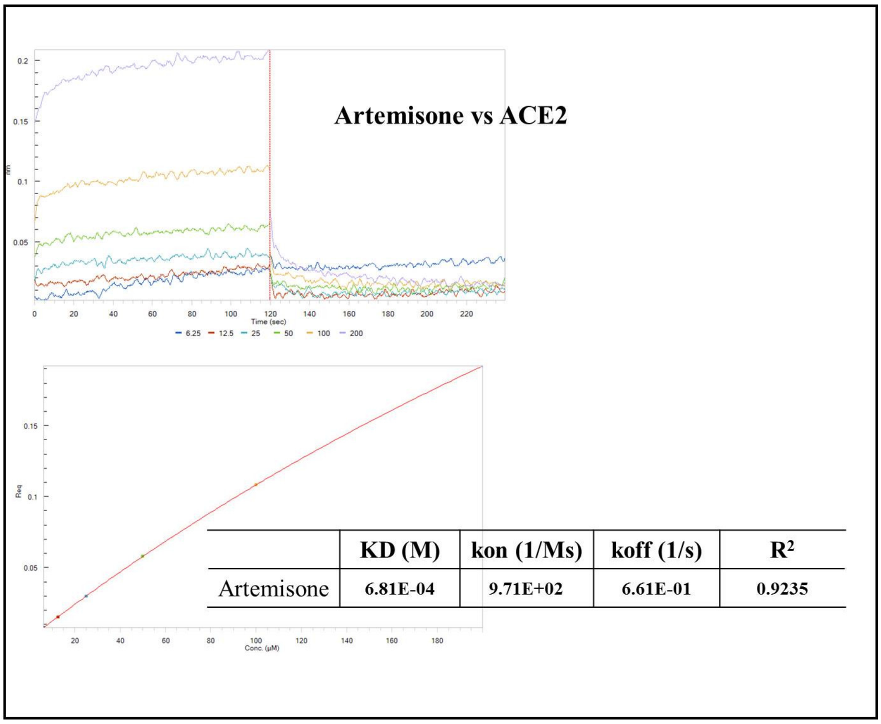

	KD (M)	kon (1/Ms)	koff (1/s)	R^2
Artemisone	**6.81E-04**	**9.71E+02**	**6.61E-01**	**0.9235**

Figure S29. Bio-layer interferometry study for the interaction of artemisone with ACE2.

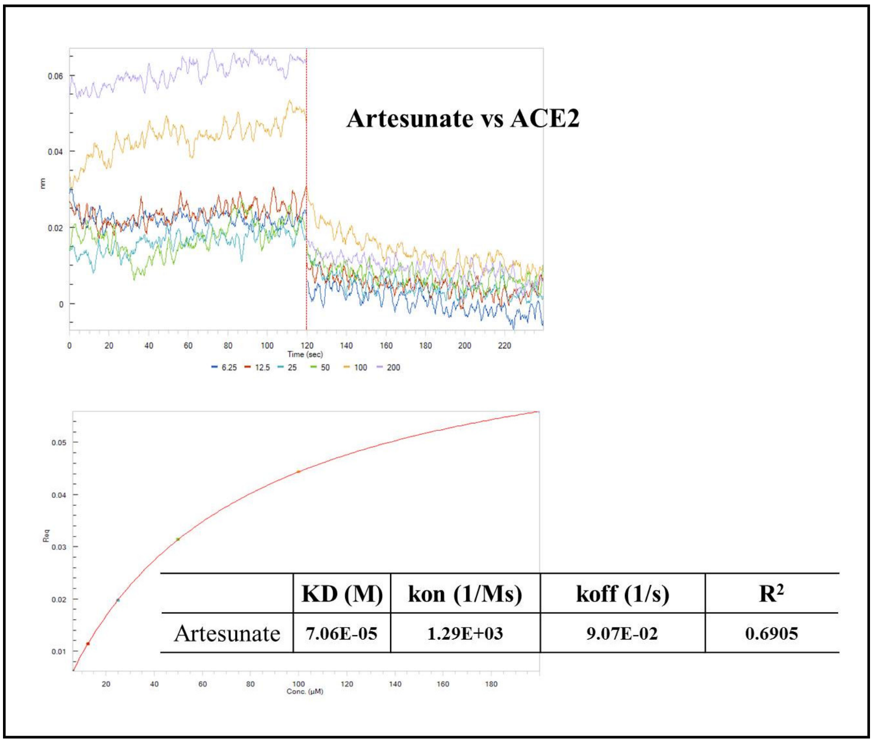

	KD (M)	kon (1/Ms)	koff (1/s)	R^2
Artesunate	**7.06E-05**	**1.29E+03**	**9.07E-02**	**0.6905**

Figure S30. Bio-layer interferometry study for the interaction of artesunate with ACE2.

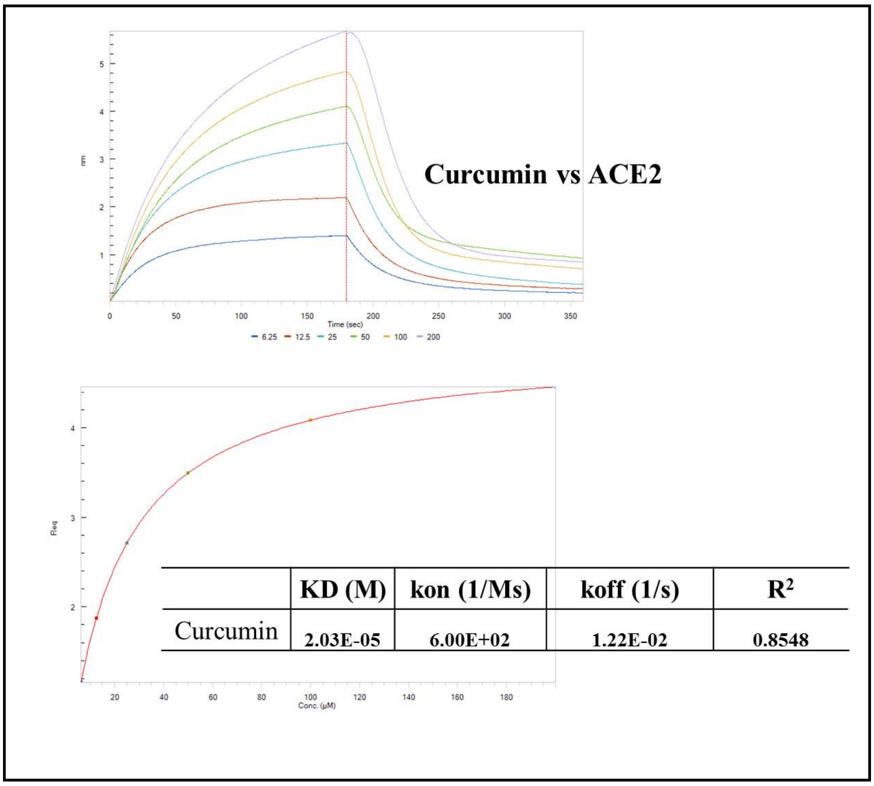

	KD (M)	kon (1/Ms)	koff (1/s)	R^2
Curcumin	2.03E-05	6.00E+02	1.22E-02	0.8548

Figure S31. Bio-layer interferometry study for the interaction of curcumin with ACE2.

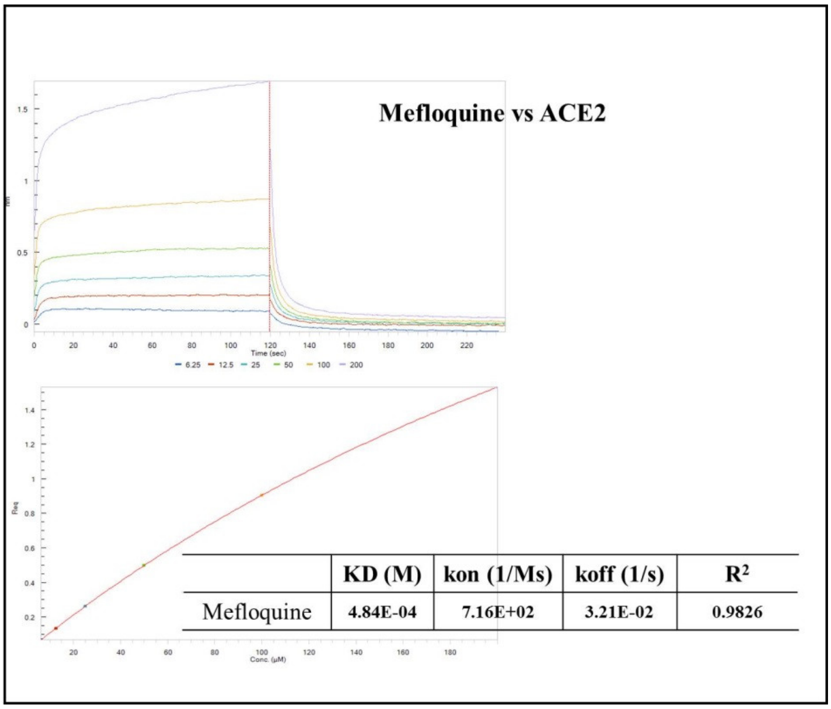

	KD (M)	kon (1/Ms)	koff (1/s)	R^2
Mefloquine	4.84E-04	7.16E+02	3.21E-02	0.9826

Figure S32. Bio-layer interferometry study for the interaction of mefloquine with ACE2.

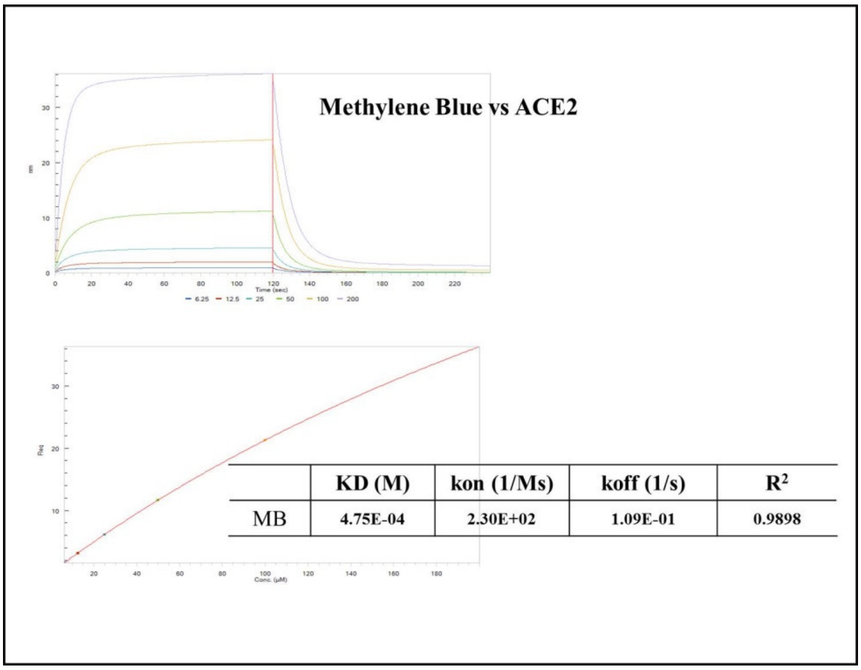

	KD (M)	kon (1/Ms)	koff (1/s)	R^2
MB	4.75E-04	2.30E+02	1.09E-01	0.9898

Figure S33. Bio-layer interferometry study for the interaction of methylene blue with ACE2.

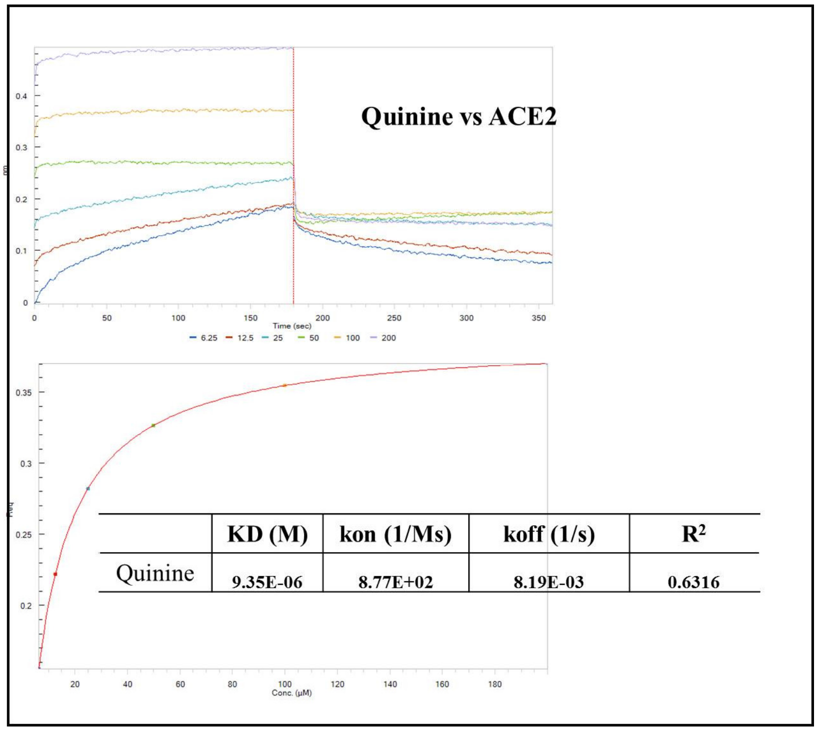

	KD (M)	kon (1/Ms)	koff (1/s)	R^2
Quinine	9.35E-06	8.77E+02	8.19E-03	0.6316

Figure S34. Bio-layer interferometry study for the interaction of quinine with ACE2.
